# Identification of a common mesenchymal stromal progenitor for the adult haematopoietic niche

**DOI:** 10.1038/ncomms13095

**Published:** 2016-10-10

**Authors:** Xingbin Hu, Mayra Garcia, Lihong Weng, Xiaoman Jung, Jodi L. Murakami, Bijender Kumar, Charles D. Warden, Ivan Todorov, Ching-Cheng Chen

**Affiliations:** 1 Divison of Hematopoietic Stem Cell and Leukemia Research, Gehr Family Center for Leukemia Research, Beckman Research Institute of City of Hope, Duarte, California 91010, USA; 2 Department of Transfusion Medicine, Xijing Hospital, Fourth Military Medical University, Xi'an 7100032, PR China; 3 Irell & Manella Graduate School of Biological Sciences, City of Hope, Duarte, California 91010, USA; 4 Bioinformatics Core, Department of Molecular Medicine, Beckman Research Institute of City of Hope, Duarte, California 91010, USA; 5 Department of Diabetes and Metabolic Diseases Research, Beckman Research Institute of City of Hope, Duarte, California 91010, USA

## Abstract

Microenvironment cues received by haematopoietic stem cells (HSC) are important in regulating the choice between self-renewal and differentiation. On the basis of the differential expression of cell-surface markers, here we identify a mesenchymal stromal progenitor hierarchy, where CD45^−^Ter119^−^CD31^−^CD166^−^CD146^−^Sca1^+^(Sca1^+^) progenitors give rise to CD45^−^Ter119^−^CD31^−^CD166^−^CD146^+^(CD146^+^) intermediate and CD45^−^Ter119^−^CD31^−^CD166^+^CD146^−^(CD166^+^) mature osteo-progenitors. All three progenitors preserve HSC long-term multi-lineage reconstitution capability *in vitro*; however, their *in vivo* fates are different. Post-transplantation, CD146^+^ and CD166^+^ progenitors form bone only. While Sca1^+^ progenitors produce CD146^+^, CD166^+^ progenitors, osteocytes and CXCL12-producing stromal cells. Only Sca1^+^ progenitors are capable of homing back to the marrow post-intravenous infusion. Ablation of Sca1^+^ progenitors results in a decrease of all three progenitor populations as well as haematopoietic stem/progenitor cells. Moreover, suppressing production of KIT-ligand in Sca1^+^ progenitors inhibits their ability to support HSCs. Our results indicate that Sca1^+^ progenitors, through the generation of both osteogenic and stromal cells, provide a supportive environment for hematopoiesis.

## Abstract (web summary)

How the environment of the niche regulates haematopoietic stem cells (HSC) is unclear. Here, the authors identify a mesenchymal stromal progenitor hierarchy and identify Sca1+ cells as common progenitors for mesenchymal stromal cells in the adult niche that provide a supportive environment for hematopoiesis.

## Graphical abstract

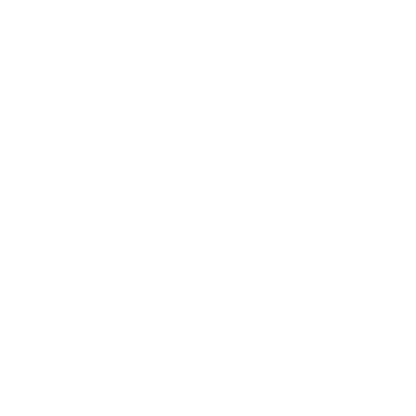

Haematopoietic stem cells (HSCs) reside in highly specific bone marrow (BM) microenvironments (known as niches) that regulate their survival, proliferation and differentiation. Both intrinsic and extrinsic regulatory cues are integrated within the niche to maintain effective control over HSCs, ensuring they support hematopoiesis without inducing aberrant proliferation^1^,^2^,^3^.

Many studies have investigated the cellular compositions and anatomical site(s) of hematopoietic niches. Osteoblasts, endothelial cells, adipocytes and several variants of perivascular stromal cells including the CD146-expressing cells in humans, nestin^+^ mesenchymal stromal cells (MSCs), leptin receptor-expressing mesenchymal cells, Mx1^+^ stromal cells and CXCL12-abundant reticular (CAR) cells have all been proposed to participate in the regulation of HSCs in the BM 4.

MSCs are currently defined as a cell population with colony forming capability (colony forming unit-fibroblastic, CFU-F) and the ability to undergo osteogenic, chondrogenic and adipogenic differentiation *in vitro*^5^,^6^. An obstacle to understanding the role of MSCs in the HSC niche is the heterogeneous nature of the MSC population and the need to identify functionally distinct subgroups. Studies have addressed this problem by identifying markers such as Osterix, LepR, Nestin, Sca1, CD146 and CD166 that can distinguish MSC subtypes^4^, but a functional hierarchy that defines a lineage of early and late progenitors has not been identified. Therefore, it is difficult to assess the relationships within the niche cell population as well as the individual contributions of different cell types to support HSCs.

We have established an *in vivo* ectopic bone-forming assay in which the cellular and molecular components of the HSC niche can be genetically modified and explored. In this system, fetal bone cells are introduced under the kidney capsule, a highly vascularized region known to support tissue engraftments. Using this assay, we identified a fetal osteochondral progenitor as the HSC niche-initiating cell^7^. A recent fate-mapping study showed that the fetal niche-initiating cells and adult niche maintenance cells are distinct; they found that LepR^+^ mesenchymal stromal cells arise postnatally and give rise to bone and adipocyte cells in the adult bone marrow^8^. Here, we identify markers that can subdivide the mesenchymal stromal cell population into early and late progenitors that are functionally distinct. Using the *in vivo* ectopic bone-forming assay, we identified a mesenchymal stromal progenitor hierarchy in the BM: CD45^−^Ter119^−^CD31^−^CD166^−^CD146^−^Sca1^+^ (Sca1^+^) cells are the most primitive, giving rise to intermediate progenitors CD45^−^Ter119^−^CD31^−^CD166^−^CD146^+^ (CD146^+^) and mature osteo-progenitors CD45^−^Ter119^−^CD31^−^CD166^+^CD146^−^ (CD166^+^). All three progenitors display the characteristics of mesenchymal stromal cells and posses the ability to support hematopoiesis *in vitro*. Their differentiation potential and ability to traffic back to the BM *in vivo* varies. CD146^+^ and CD166^+^ progenitors form only bone *in vivo*; in contrast, Sca1^+^ cells differentiate into CXCL12-producing stromal cells, CD146^+^ intermediate mesenchymal progenitors and CD166^+^ mature osteo-progenitors. Ablation of Sca1^+^ cells results in a decrease of CD146^+^ and CD166^+^ cells, supporting our hierarchical designation; as well as a reduction in Lineage^−^Sca1^+^ckit^+^ (LSK) cells and long-term HSCs (LT-HSCs; LSK-CD150^+^CD48^−^CD135^−^). Using shRNA knockdown, we show that KIT-ligand (KITL) gene expression in Sca1^+^ progenitor derived cells is indispensable for maintaining HSCs. Our findings suggest that Sca1^+^ progenitors act as a common progenitor of mesenchymal stromal cells in the adult niche.

## Results

### Identification of three mesenchymal stromal progenitors

**Figure 1:**
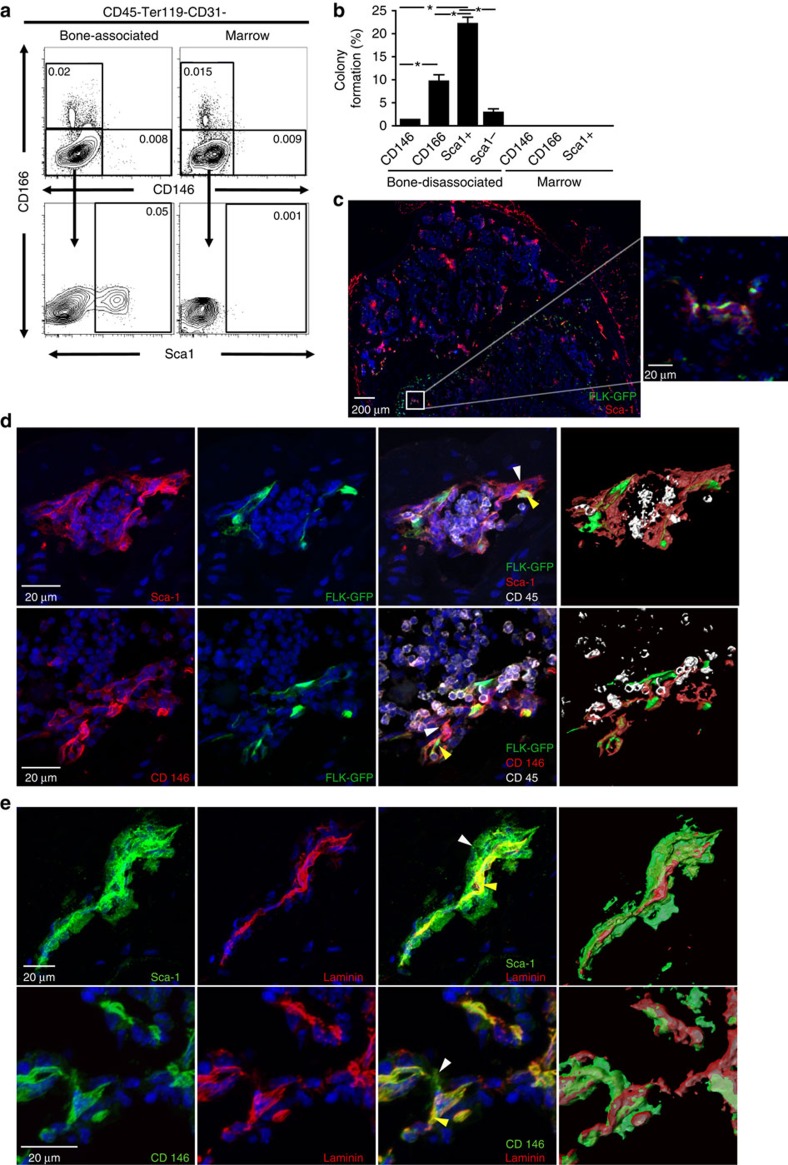
Phenotypic Identification of three bone-disassociated mesenchymal progenitors. (**a**) Representative FACS profiles of stromal cells (CD45^−^Ter119^−^CD31^−^) near the endosteum (bone-disassociated). They were separated based on their CD166, CD146 and Sca1 expression profiles. Numbers shown in the gate represent the percentage of total live cells. (**b**) Single-cell colony forming efficiency of BM stromal progenitors (*:*P*≤0.05, three repeats of 60 cells each, student's *t*-test). (**c**) Sections of femur from Flk1-GFP mouse stained with antibodies against Sca1 and scanned with an iCys Research imaging cytometer. Nuclei were stained with Dapi. (**d**) Confocal maximum intensity projections of sections of femurs from Flk1-GFP mice stained with antibodies against Sca1 or CD146; scanned near the epiphyseal plate. (**e**) Confocal maximum intensity projections of sections of femur stained with antibodies against Sca1 or CD146 and laminin; scanned near epiphyseal plate. White arrows point to mesenchymal progenitors yellow arrows point to endothelial cells.

Although fetal CD105^+^Thy1.1^−^ osteochondral progenitors formed ectopic bone with marrow under the kidney capsule^7^, the phenotypically similar adult CD105^+^Thy1.1^−^ populations formed only bone without marrow after transplantation (Supplementary Fig. 1a,b). This result suggests that adult stromal progenitors may have different developmental potentials for niche formation and/or different surface markers compared with their fetal counterparts, therefore further analysis is needed to find an adult common progenitor. We subdivided the stromal population by first separating mouse marrow from crushed bone. A large portion of the HSCs and their supporting niche cells reside in the trabecular bone region^9^,^10^. To isolate the supporting niche cells, it is necessary to disassociate them from crushed bone using collagenase digestion. We designated these cells as the bone-disassociated stroma fraction, which may be part of either or both the endosteal and the perivascular niches. We then removed haematopoietic and endothelial cells by selecting the CD45^−^Ter119^−^CD31^−^ fraction (Supplementary Fig. 1c). We next subdivided this population based upon cell-surface expression of CD146, a marker for human osteoprogenitors^11^, CD166, an adhesion molecule expressed by both HSCs and MSCs^12^, and Sca1, a marker expressed by haematopoietic progenitors. Flow cytometry analysis revealed that the distributions of CD146^+^ and CD166^+^ stromal cells were similar between bone-disassociated stroma and marrow fractions (Fig. 1a). However, the frequency of Sca1^+^ cells was noticeably lower in the marrow compartment, 0.004±0.00002% versus 0.056±0.007% (Fig. 1a and Supplementary Fig. 1d). Furthermore, most of the Sca1^+^ cells were located at the epiphysis, not diaphysis (Supplementary Fig. 1e,f). To determine the colony forming potential of each stromal subset, single stromal cells were sorted and cultured for 14 days. We observed colony forming activities, from bone-disassociated cells but not marrow-derived cells (Fig. 1b). Although the Sca1^+^ cells had the highest colony forming ability, cultured Sca1^+^, CD146^+^ and CD166^+^ cells all demonstrated slightly different, but very typical MSCs morphology (Supplementary Fig. 1g). The Sca1^−^ population while more abundant (0.388±0.058% versus 0.056±0.007%) had lower colony forming ability compared with Sca1^+^ cells (Fig. 1b).

Immunofluorescence staining of femur cross sections using Sca1 antibodies revealed that most Sca1^+^ cells are localized close to the endosteal surface in the trabecular bone region, especially in the lower part of the epiphyseal plate (Fig. 1c). Staining for Sca1 in Flk1-GFP transgenic mice, in which all the endothelial cells express GFP, revealed that many Sca1^+^ stromal cells were located adjacent to the vasculature within the trabecular bone region (Fig. 1c,d). Three-dimensional visualization of confocal *z*-stacks illustrated that Sca1^+^ and CD146^+^ stromal cells, while closely associated with endothelial cells, were distinct cells (Fig. 1d) white arrows point to stromal cells, yellow arrows point to endothelial cells. To distinguish Sca1^+^ HSCs from Sca1^+^ stromal cells, haematopoietic cells were stained with an antibody against CD45, this highlighted a distinct population of CD45^−^Sca1^+^ perivascular cells that were wrapped around the endothelial cells (Fig. 1d). Co-staining with laminin, a protein abundant in basement membranes that encases the BM sinusoidal vessels and perivascular cells^13^ confirmed that Sca1^+^ cells are largely localized in the perivascular region (Fig. 1e). CD146^+^ cells were also located in the perivascular region and had similar distribution as Sca1^+^ cells (Fig. 1d,e). CD166^+^ cells, unlike Sca1^+^ and CD146^+^ cells, were mainly localized to the endosteal surface and not associated with the vasculature (Supplementary Fig. 1h).

### Sca1^+^ progenitors contribute to BM stroma formation

**Figure 2:**
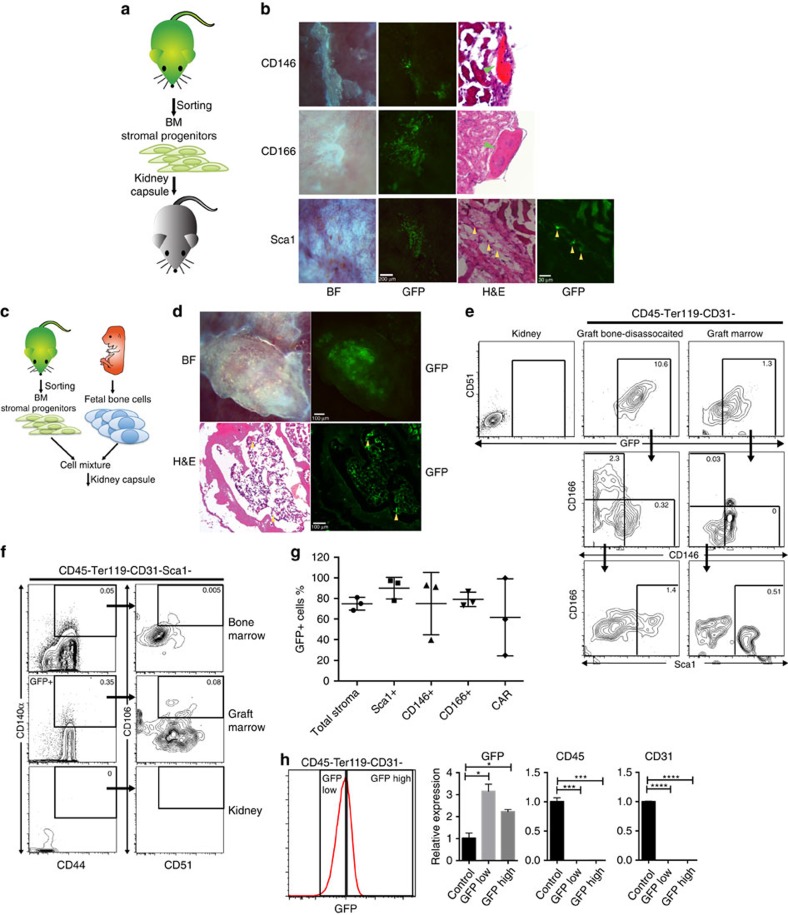
Sca1^+^ progenitors contribute to BM stroma, while CD146^+^ and CD166^+^ progenitors form bones. (**a**) Direct transplants of GFP- labelled progenitors under the kidney capsule. (**b**) Bright-field and GFP images of GFP-labelled progenitors 1 month after transplant (far left and left). A representative cross-section of the graft site was stained with H&E (right, green arrowhead points to bone) or GFP to identify the donor origin (far right, yellow arrowheads). (**c**) Co-tranplants of GFP-labelled adult progenitors with non-GFP fetal skeletal progenitors under the kidney capsule. (**d**) Bright-field and GFP images of GFP-labelled Sca1^+^ mixed with fetal skeletal progenitors 1 month after transplant. Donor-derived GFP^+^ cells can be clearly identified (far left and left). Representative cross sections of the graft site stained with H&E (right) or GFP ( far right) to identify the donor origin (yellow arrowheads). (**e**–**f**) Representative FACS analysis of graft of mixed GFP-labelled Sca1^+^ progenitors and non-GFP skeletal progenitors harvested 1 month after transplant. The per cent of live cells is displayed for each gate (**e**) FACS analysis of donor-derived endosteum associated progenitors (left) and marrow stromal cells (right). (**f**) FACS analysis for phenotypically defined CAR cells in control bone marrow, marrow of graft and kidney. (**g**) Percentage of GFP+ cells for each group; mean±s.d. (*n*=3). Total stroma is the Ter119-CD45-CD31- population. (**h**) Histrogram for GFP to show the populations that were sorted for RNA isolation (far left). Relative expression of GFP, CD45 and CD31 in control cells (untransplanted kidney), GFP low gate cells and GFP high gate cells (*P<0.05, ***P<0.0005, ****P<0.00005, mean±s.d., technical triplicate, student's *t*-test.

To determine if the adult mesenchymal progenitors possessed similar niche-initiating capabilities to the fetal osteochondral progenitors, we transplanted bone-disassociated CD146^+^, CD166^+^ or Sca1^+^ progenitors under the mouse kidney capsule (Fig. 2a). One month later we observed no marrow formation from these grafts. The CD146^+^ and CD166^+^ progenitors produced bone, whereas the Sca1^+^ cells produced a limited fibroblast-like cell growth (Fig. 2b). This suggests that CD146^+^ and CD166^+^ cells are likely osteo-progenitors committed to an osteoblastic fate, while Sca1^+^ cells may be more primitive, and require signals not present in the kidney capsule in order to differentiate into a specific cell type. Moreover, although all three stromal subsets have classically defined MSC characteristics, the Sca1^+^ cells can be distinguished by their *in vivo* differentiation potential.

The *in vivo* relationship with other niche cells can potentially alter stromal cell differentiation. To model the multiple cell populations in the developing niche we co-transplanted GFP-expressing bone-disassociated adult progenitors, isolated from C57BL/Ka-Thy1.1-CD45.1-GFP mice, with unmarked fetal skeletal progenitors under the kidney capsule (Fig. 2c). Progeny of CD146^+^ and CD166^+^ progenitors could only be found in the bone portion of the graft, and not in the marrow area of the graft (Supplementary Fig. 2a,b). The Sca1^−^ cells failed to contribute to the graft evidenced by the lack of GFP^+^ cells (Supplementary Fig. 2c). In contrast, Sca1^+^ progenitor derived cells could clearly be identified in the area beneath the bone (Fig. 2d). A cross-section of the graft revealed that donor-derived GFP^+^ cells mainly localized within the marrow compartment and had a reticular cell-like structure, with some cells encircling the vasculature (Fig. 2d and Supplementary Fig. 2g). Staining with anti-GFP antibody confirmed that the Sca1^+^-derived cells were located within the marrow portion of the bone graft (Supplementary Fig. 2d). Flourescent-activated cell sorting (FACS) analysis of the kidney graft indicated that Sca1^+^ progenitors gave rise to phenotypic CAR cells (61.67±.37.53%) [Sca1^−^CD44^+^CD51^+^CD106^+^CD140a^+^ (ref. 14)] and Sca1^+^ stromal cells in the marrow of the graft (Fig. 2e–g). Donor-derived CD146^+^ (75.00±30.32%), CD166^+^(79.13±6.976%) and Sca1^+^ (90.07±10.52%) cells were found in the bone-disassociated fraction of the graft (Fig. 2e,g). The kidney cells are highly auto-fluorescent thus we stained kidney cells and used them as a negative control to set the GFP gate (Fig. 2e). When we used a more stringent gate for GFP Sca1^+^-derived CD146^+^, CD166^+^ and Sca1^+^ were still identified (Supplementary Fig. 2e). We found neither haematopoietic (CD45^+^) nor endothelial (CD31^+^) contributions from the transplanted GFP^+^ cells by immunostaining of graft sections, suggesting that the Sca1^+^GFP^+^ transplanted cells were a pure population (Supplementary Fig. 2f,g). Furthermore, we show by FACS analysis that the CD45^+^ cells found in the kidney transplanted with Sca1^+^GFP^+^ cells are negative for GFP and the CD45^−^Ter119^−^CD31^−^ cells are positive for GFP (Supplementary Fig. 3a,b). We sorted CD45^−^Ter119^−^CD31^−^ GFP low and GFP high cells from a kidney transplanted with Sca1^+^GFP^+^ cells and found that they expressed GFP by quantitative real-time PCR (qRT-PCR), while cells from a control untransplanted kidney did not (Fig. 2h). Furthermore, the control cells, which were mixed population of stromal, haematopoietic and endothelial cells, had high expression of CD45 and CD31. The sorted GFP^+^ cells, in contrast, had undetectable levels of CD45 and CD31 suggesting that the transplanted cells are not derived from and do not give rise to haematopoietic or endothelial cells (Fig. 2h). Similar results were obtained after cells from a clone of a single Sca1^+^ progenitor were transplanted with fetal cells or alone under the renal capsule, this confirmed that the various cell types were derived from a single progenitor and not from a mixed population within the Sca1^+^ sorted progenitors (Supplementary Fig. 3c,d). These results support a view where Sca1^+^ cells are primitive mesenchymal stromal progenitors, while CD146^+^ and CD166^+^ are more mature osteo-progenitors as they were only found to give rise to bone cells.

**Figure 3:**
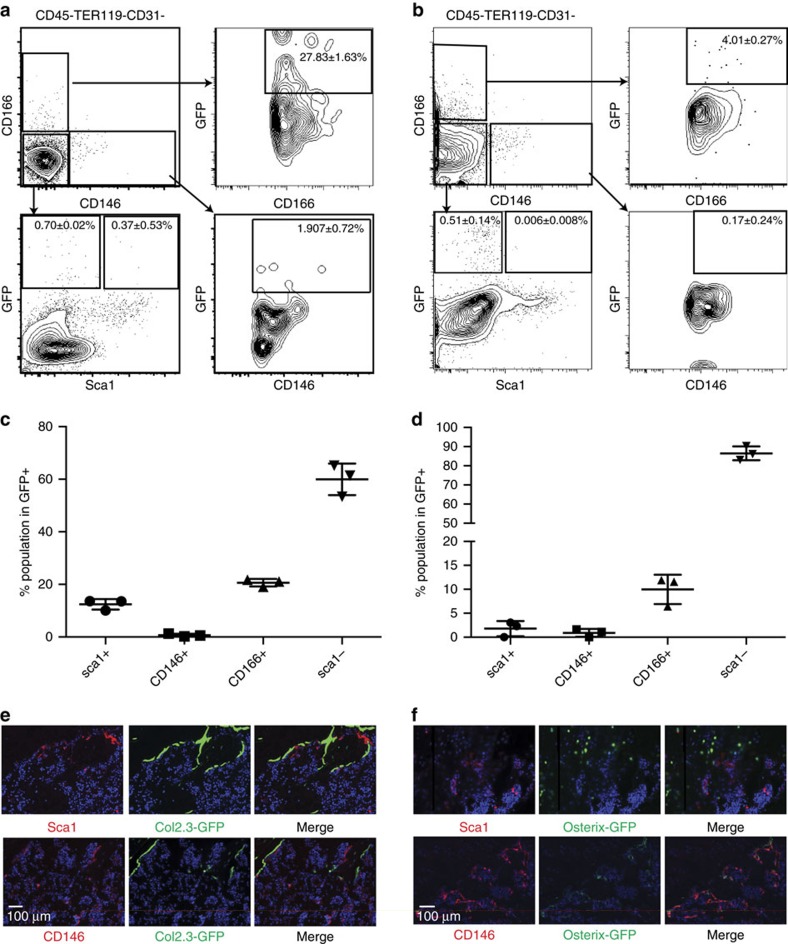
CD 146^+^ and CD 166^+^ cells were marked by Col2.3-GFP or Osx1-Cre:GFP while Sca1^+^ cells were not. (**a**–**b**) FACS analysis of bone-disassociated stromal cells (CD45^−^TER119^−^CD31^−^) from (**a**) Col2.3-GFP or (**b**) Osx1-Cre:GFP mice. The fequency of the parent gate is displayed. (**c**–**d**) The frequency of Sca1+, CD146+, CD166+ and Sca1- in the GFP+ population for (mean±s.d.) (**c**) Col2.3-GFP and (**d**) Osx1-Cre:GFP obtained from FACS analysis (*n*=3). (**e**–**f**) Sections of femurs from (**e**) Col2.3-GFP or (**f**) Osx1-Cre:GFP mice stained with antidodies to Sca1 or CD146.

The fact that CD146^+^ and CD166^+^ stromal subsets were only found in the bone portion of the graft suggests they mainly contain osteoblast progenitors. This is further supported by our analysis of BM stromal cells in Osx1-Cre:GFP and Col2.3-GFP transgenic mice, in which osteo-progenitors and mature osteoblasts, respectively, are marked by GFP expression^15^,^16^. In Col2.3-GFP mice, more bone-disassociated CD166^+^ (27.83±1.63%) cells than CD146^+^ (1.91±0.72%), Sca1^+^ (3.55±1.18%) and Sca1^−^ (1.82±0.68) expressed GFP (Fig. 3a). Because the Sca1^−^ population is more abundant the majority of GFP-expressing cells were Sca1^−^ (Fig. 3c). In the Osx1-Cre;GFP mice the majority of GFP-expressing cells were also Sca1^−^ (Fig. 3d), although the frequency of GFP was hig her in the CD166^+^ population than in the Sca1^−^ population (4.01±0.27% versus 0.41±0.005%) (Fig. 3b). This suggests that CD166^+^ cells are more enriched for osteo-progenitors and osteoblast. Immunofluorescence staining revealed that most Sca1^+^ cells did not co-localize with the GFP^+^ cells in these animals (Fig. 3e,f). All together, the results suggest that CD146^+^, CD166^+^ and Sca1^+^ progenitors function differently *in vivo*. Sca1^+^ cells were the most primitive of all three progenitors; they preserved themselves after *in vivo* transplantation and gave rise to multiple cell types (Fig. 2e–g), and had a high cloning efficiency (Fig. 1b), while CD146^+^ and CD166^+^ appear to be osteo-progenitors.

### Only Sca1^+^ progenitors home to BM after intravenous infusion

**Figure 4:**
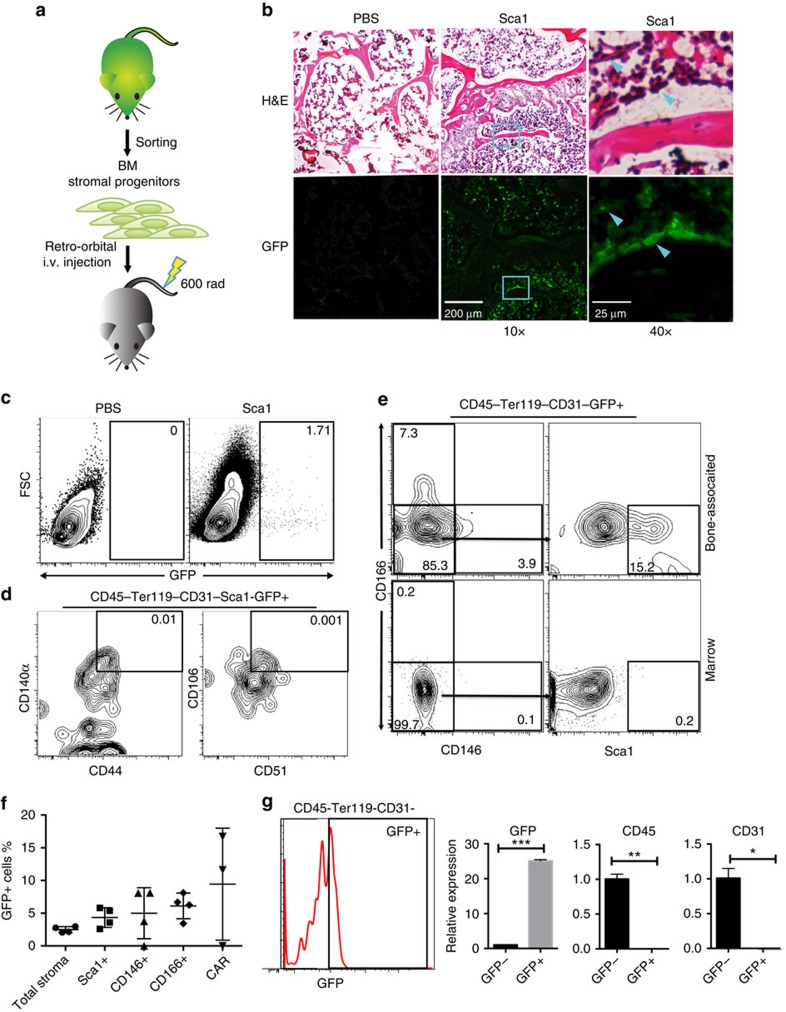
Sca1^+^ progenitors hometo the BM after intravenous transplantation into irradiated mice. (**a**) Intravenous transfusion of GFP-lablled mesenchymal progenitors into irradiated mice (600 rad). (**b**) Representative cross sections of the tibia stained with H&E (upper) or GFP (lower). Tibias were harvested 1 month after transplantation. Blue arrows point to GFP+ stromal cells (**c**–**d**) FACS analysis of the marrow of recipients injected with Sca1^+^ cells for (**c**) GFP expression or (**d**) phenotypes of CAR cells. (**e**) FACS analysis of donor-derived bone-disassociated progenitors (upper) and marrow stromal cells (lower). (**f**) Frequency of GFP+ cells in each group; mean±s.d. (*n*=4). (**g**) Histrogram of GFP to show the population that was sorted for RNA isolation (far left). Relative expression of GFP, CD45 and CD31 in GFP- and GFP+ sorted cells from CD45^−^Ter119^−^CD31^−^ stromal cell gate (*P<0.05, **P<0.005, ***P<0.0005, mean±s.d., technical triplicate, student's *t*-test).

We next tested the physiological role of bone-disassociated mesenchymal progenitors in their endogenous BM microenvironment. To evaluate the homing ability of CD146^+^, CD166^+^, Sca1^−^ and Sca1^+^ progenitors, we injected freshly isolated GFP-expressing progenitors from C57BL/Ka-Thy1.1-CD45.1-Tg(CAG-GFP) mice intravenously into sub-lethally irradiated mice (Fig. 4a). One month later, we identified donor-derived stromal cells only in the BM of animals that received Sca1^+^ progenitors (Fig. 4b,c). No donor-derived tissue could be found in animals that received PBS, CD146^+^, CD166^+^ or Sca1^−^ cells, by both immunohistochemistry and FACS analysis (Supplementary Fig. 4a). FACS analysis revealed that intravenously transferred Sca1^+^ progenitors gave rise to cells with surface markers of CAR (9.43±8.558%) cells in the marrow fraction and CD146^+^ (4.99±3.993%), CD166^+^ (6.11±1.959%) and Sca1^+^ (4.34±1.507%) cells in the bone-disassociated fraction (Fig. 4d–f). To exclude the possibility that the GFP^+^ cells observed in the Sca1^+^ IV transplanted mice were derived from HSCs, we stained sections with anti-CD45 and CD41 antibodies to show that GFP did not overlap with these markers (Supplementary Fig. 4b). FACS analysis also revealed that CD45^+^ and CD31^+^ cells in the Sca1^+^ IV transplanted mice do not express GFP, while CD45^−^Ter119^−^CD31^−^ cells do (Supplementary Fig. 4c). qRT-PCR on the sorted bone-dissociated CD45^−^Ter119^−^CD31^−^GFP^+^ and GFP^−^ cells isolated 1 month post IV-injection verified the GFP expression and showed that while the GFP^−^ cells expressed CD45 and CD31, the GFP^+^ cells did not, excluding the possibility that GFP^+^ cells are derived from haematopoietic or endothelial lineages (Fig. 4g). Moreover the stromal nature of the GFP+ cells is illustrated in the hematoxylin and eosin (H&E) stained section (Fig. 4b, blue arrows).

One of the key defining markers for CAR cells is the expression of the chemokine CXCL12 (ref. 17). We performed qRT-PCR to determine the CXCL12 expression levels in Sca1^+^ progenitors and their progeny. Sca1^+^ progenitors expressed CXCL12 at a low level compared with marrow CAR cells. However, after transplantation, Sca1^+^ progenitors gave rise to progeny that expressed CXCL12 at comparable levels to CAR cells in marrow, both in the kidney graft and IV transplantation (Supplementary Fig. 5a). Furthermore, we could detect CXCL12-producing, phenotypic CAR cells even when Sca1^+^ cells were transplanted under the kidney capsule without fetal cells, suggesting that differentiation of Sca1^+^ progenitors into CAR cells may be a default state whereas environmental signals are necessary for the osteogenic fate (Supplementary Fig. 5a,b). To exclude the possibility that the observed donor-derived cells were a result of transplanted Sca1^+^ progenitors fused with host cells, we injected GFP^+^ Sca1^+^ cells into sub-lethally irradiated mice that constitutively express the tdTomato fluorescent protein (tdTomato). We did not detect any cells that co-expressed GFP and tdTomato. These data suggest that the donor contributions arose from the proliferation and differentiation of transplanted Sca1^+^ progenitors and not from fusion with host cells (Supplementary Fig. 5c).

Taken together, our results demonstrate that, among the three mesenchymal progenitors, only Sca1^+^ progenitors can home back to the BM and that they contribute to the formation of both bone and the BM microenvironment. This result supports our conclusion that Sca1^+^ cells represent the most primitive progenitor and are capable of giving rise to CD146^+^, CD166^+^ cells and CAR cells.

### Mesenchymal progenitors express HSC supporting genes

**Figure 5:**
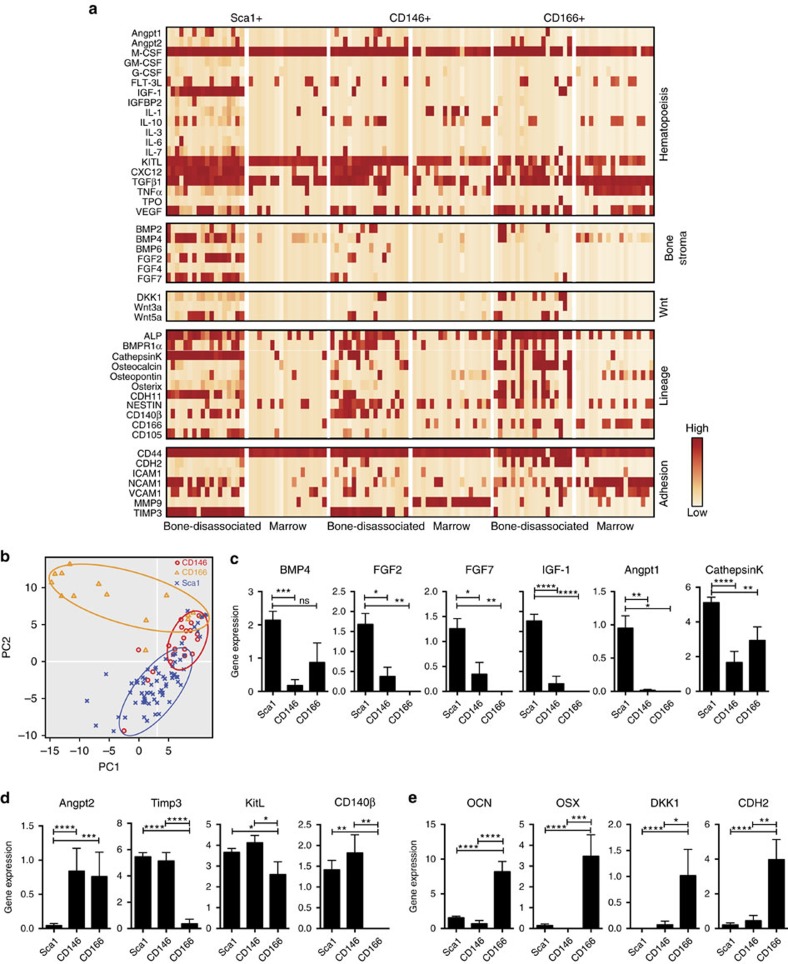
Bone-disassociated mesenchymal progenitors and marrow stromal cells possess different gene expression profiles. (**a**) Representative qRT-PCR of 18 single cells from CD146^+^, CD166^+^ or Sca1^+^ stromal populations either near the endosteum (bone-disassociated) or in the marrow (Marrow) using the Fluidigm 48.48 dynamic array. Each column within a given population represents expression in an individual cell. (**b**) Comparison of the first and second prinicpal components from a principal component analysis of single-cell PCR gene expression data from all 47 genes. (**c**–**e**) Expression patterns of genes that are significantly overexpressed in mean±s.d. (**c**) Sca1^+^ cells (**d**) Sca1^+^ cells and/or CD146^+^ cells, or (**e**) CD166^+^ cells. Statistical significance was calculated using one-way ANOVA analysis *P≤0.05, **P≤0.005, ***P≤0.00; *n*=72. ANOVA, analysis of variance.

To understand the molecular mechanisms underlying the functional differences among the Sca1^+^ mesenchymal stromal progenitor and CD146^+^ and CD166^+^ osteo-progenitors, we performed single-cell multi-gene qRT-PCR to measure the expression levels of 47 niche-related genes and the housekeeping gene, *GAPDH*. In agreement with our CFU-F studies, and despite the expression of similar surface markers, the bone-disassociated progenitors and marrow-localized cells had remarkably different gene expression profiles; the former expressed a wider repertoire of genes related to the support of HSC functions (Fig. 5a). On the basis of only the gene expression values, principal component analysis partitioned the bone-disassociated cells by their surface marker phenotype (Fig. 5b). Principal component 1, which captures the largest proportion of the variation in the data, separates the Sca1^+^ and CD146^+^ cells from the CD166^+^ cells. Principal component 2 further separates the Sca1^+^ and CD146^+^ cells (Fig. 5b). However, although individual populations can be distinguished, there is also some overlap, particularly between the Sca1^+^/CD146^+^ and CD146^+^/CD166^+^ cells. These data suggest that CD146^+^ cells may be acting as an intermediary osteo-progenitor. We compared the gene expression values among the three bone-disassociated progenitors and identified statistically significant variations in expression levels of shared genes. Sca1^+^ progenitors expressed higher levels of cytokines that are important for skeletal development (*BMP4, FGF2, FGF7 and IGF-1*) and HSC quiescence (*Angpt1*). They also expressed cathepsin K, an enzyme important for bone remodelling and absorption^18^ (Fig. 5c). CD146^+^ cells expressed *Angpt2*, an antagonist of *Angpt1*, at higher levels than Sca1^+^ and CD166^+^ cells. Both Sca1^+^ and CD146^+^ cells expressed *TIMP3*, an endogenous metalloproteinase that can recruit quiescent HSCs into the cell cycle^19^, and *KITL*, a key player in maintaining HSCs, at higher levels than the CD166^+^ cells (Fig. 5d). Consistent with our immunofluorescence studies (Fig. 1d,e), bone-disassociated Sca1^+^ and CD146^+^ cells also expressed high levels of CD140β, a common marker for perivascular cells^20^ (Fig. 5a,d). In agreement with our transgenic mouse (Fig. 3d) and transplantation studies (Fig. 2b), expression of osteocalcin, the mature osteoblast marker, was highest in bone-disassociated CD166^+^ cells and lower in Sca1^+^ and CD146^+^ cells, suggesting CD166^+^ cells are more differentiated than both Sca1^+^ and CD146^+^ cells (Fig. 5a,e). CD166^+^ progenitors also expressed other osteo-lineage markers including *Osterix* and *DKK1*, a Wnt signalling inhibitor that suppresses bone formation^21^, at higher levels than Sca1^+^ and CD146^+^ cells (Fig. 5a,e). Taken together, these data support a model where the CD166^+^ cells are the most mature and the Sca1^+^ progenitors are the most primitive with CD146^+^ possibly acting as an intermediary.

Nestin- and Leptin receptor-expressing mesenchymal stromal cells were previously shown to be important components of the HSC niche^8^,^22^,^23^. Interestingly, although *nestin* expression was identified in most of the bone-disassociated CD146^+^ and CD166^+^ cells, only a small portion of the Sca1^+^ cells expressed *nestin* (Fig. 5b). Indeed, when we analysed the BM stromal cells in nestin-GFP transgenic mice, we found only a few cells in the marrow and bone-disassociated fractions that expressed both Sca1 and Nestin together (Supplementary Fig. 6a,b). Suggesting that Nestin expression may be a characteristic of more differentiated stromal cell types. Using the leptin receptor (LepR) as a maker, Sean Morrison's group identified a perivascular stromal population that is instrumental to HSC maintenance and can give rise to bone and adipose cells in the adult bone marrow^8^,^23^. We observed LepR antibody staining in 0.41±0.040% of live cells and 70.47±1.78% of the CD31-Ter119-CD45- stromal population (Supplementary Fig. 6c). The highest levels of LepR antibody staining were found in the CD166^+^ (90.2±6.67%) subpopulation compared with Sca1^+^ (74.9±5.86%), CD146^+^ (80.8±8.63%) and Sca1^−^ (66.73±2.20%) subpopulations (Supplementary Fig. 6d). Consistent with the antibody staining the mesenchymal stromal cell populations showed similar relative expression by qPCR, using a probe that recognizes all four transcripts of *LepR* (Supplementary Fig. 6e). Together these data suggest that Sca1, CD146 and CD166 could be used to subdivide the LepR-expressing mesenchymal cells, providing a more detailed view of the HSC niche.

### Mesenchymal progenitors are important for HSC maintenance

**Figure 6:**
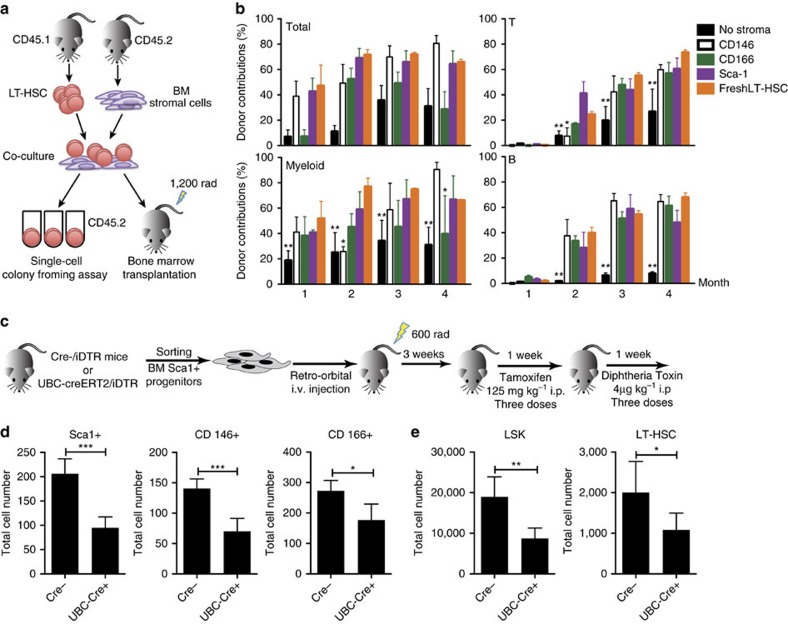
Bone-disassociated mesenchymal progenitors support HSC maintenance *in vitro*. (**a**) LT-HSCs were cultured with mesenchymal progenitors for two days, then analysed for single-cell conlony forming ability or competitive repopulation. (**b**) Contribution of donor HSCs in the blood assayed 1–4 months after transplantation (*n*=8). (**c**) Sca1^+^ mesenchymal progenitors from Cre^−^/iDTR or UBC-creERT2/iDTR mice were i.v. injected into mice and then ablated by tamoxifen injection. (**d**–**e**) FACS analysis of (**d**) the mesenchymal stromal progenitor population and (**e**) the LSK and LT-HSC populations directly after ablation of Sca1^+^ mesenchymal stromal cells Cre^−^ *n*=4 and Cre^+^ *n*=5 from two combined experiments. (For **b** and **e**; *P≤0.05, **P≤0.005, ***P≤0.001, mean±s.d., student's *t*-test).

To evaluate the individual ability of the identified bone-disassociated progenitors to support HSCs, we co-cultured sorted LT-HSCs with each progenitor. After two days, we plated the co-cultured HSCs as single cells per well in a cytokine cocktail that promotes full erythromyeloid potential. We then analysed individual wells by flow cytometry at day 7 and day 14 to determine lineage potential of cultured HSCs (Fig. 6a). HSCs co-cultured with stromal cells/progenitor cells had a higher plating efficiency than HSCs cultured without stroma. HSCs co-cultured with Sca1^+^ or CD146^+^ progenitors produced more granulocyte/macrophage (GM) lineages colonies compared with HSCs co-cultured with CD166^+^ progenitors (Supplementary Fig. 7a). To determine if cultured HSCs could functionally reconstitute multi-lineage hematopoiesis *in vivo*, we transplanted 200 cultured HSCs with 2 × 10^5^ whole bone marrow cells into lethally irradiated mice. We observed that *in vitro* co-culture with Sca1^+^ and CD146^+^ progenitors strongly promoted survival and maintenance of multi-lineage reconstitution by HSC. In contrast, there was little engraftment by HSCs previously cultured without stroma under these minimal culture conditions. Intriguingly, HSCs cultured with CD166^+^ progenitors had lower myeloid engraftment but similar B- and T-cell engraftment when compared with HSCs cultured with Sca1^+^ or CD146^+^ progenitors (Fig. 6b). It is possible that prolonged *in vitro* culture of the identified progenitors might detrimentally affect their ability to support HSCs, due to *in vitro* culture-induced apoptosis, senescence, or other changes. To test this possibility, we performed a co-culture experiment using *in vitro* expanded progenitors instead of freshly isolated ones. We observed a reduced but similar pattern of engraftment (Supplementary Fig. 7b). Our data indicate that these three progenitors were capable of maintaining HSCs and therefore possessed, at least *in vitro*, functional HSC niche activity.

To test the ability of the mesenchymal stromal progenitors to support HSCs *in vivo*, we took advantage of the Sca1^+^ cells' ability to home to the marrow and developed a system for altering the mesenchymal stromal cell population within the HSC niche. We sorted Sca1^+^ cells from mice expressing the diphtheria toxin receptor (DTR) under control of a tamoxifen-inducible promoter (UBC-creERT2/iDTR) and from matched control (Cre-/iDTR) mice^24^ and injected them into sub-lethally irradiated C57BL/Ka mice (Fig. 6c). Under normal conditions, mice are not sensitive to diphtheria toxin. However, when we injected tamoxifen 3 weeks post transplantation, DTR was expressed only in the transplanted cells. Thus, the diphtheria toxin could selectively ablate the injected UBC-creERT2/iDTR Sca1^+^ cells and any cells that were derived from them (Fig. 6c). All three stromal populations were decreased, supporting a model where CD146^+^ and CD166^+^ cells are derived from Sca1^+^ cells (Fig. 6c). We analysed the LSK and LT-HSC populations and found that the total cell number of both was significantly decreased (Fig. 6e). This suggests that the mesenchymal stromal progenitor population as a whole is necessary for proper maintenance of HSCs.

### KITL in Sca1^+^ cells is important for HSC maintenance

Single-cell qRT-PCR profiling of bone-disassociated progenitors identified several significantly different genes, including *TIMP3, CD140β, KITL, DKK1, Osterix, CD166, CDH2* and *Osteocalcin* (Fig. 5c–e and Supplementary Fig. 5d). Altered expression of these genes could contribute to the inferior ability of CD166^+^ progenitors to support LT-HSC reconstitution, compared with CD146^+^ and Sca1^+^ progenitors. For further analysis, we excluded genes that could represent differences in developmental stages (*Osterix* and *Osteocalcin*), or are known surface markers on other cell populations (*CD140β* and *CD166*), or had conflicting reports regarding their role in hematopoiesis (*CDH2*). We chose to further study *KITL* because of its well-known role in supporting HSCs.

**Figure 7:**
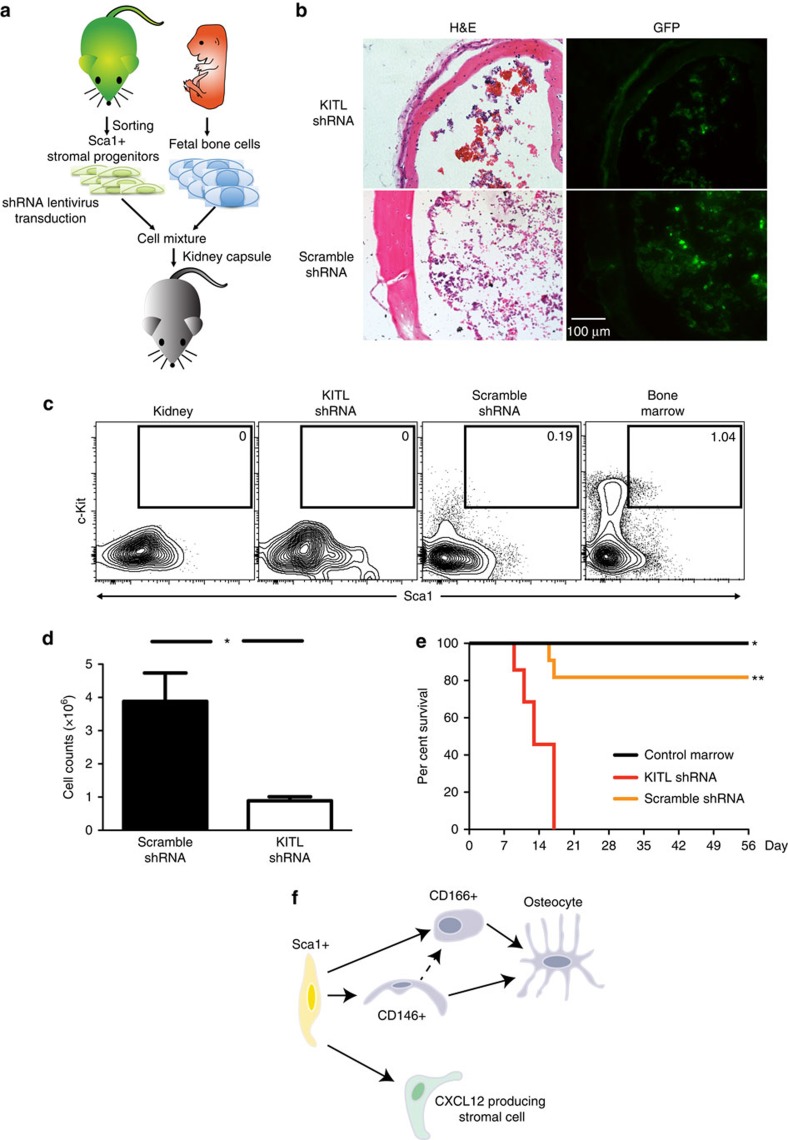
Disruption of KITL production in Sca1^+^ progenitors suppressed hematopoiesis in the ectopic niche. (**a**) Sca1^+^ progenitors were transduced with lentivirus as indicated and 1,000 sorted GFP^+^ Sca1^+^ progenitors were co-transplanted with unmarked fetal skeletal progenitors under the kidney capsule. (**b**) Representative cross sections of the engrafted ectopic bone stained with H&E (right) or GFP (left) to identify the donor origin, harvested 1 month after transplant. (**c**) Representative FACS profiles of LSK frequencies that were pre-gated for live, CD45^+^lineage^−^ cells. (**d**) Cellularity of the ectopic niche marrow (*P<0.05, *n*=5, student's *t*-test). (**e**) Rescue of lethally irradiated mice with 2 × 10^5^ marrow cells from engrafted ectopic niche generated with either mixed Sca1^+^/shKITL progenitors (red) or with Sca1^+^/Scrambled shRNA progenitors (yellow) or control bone marrow from host tibia (black) (*n*=8). (**f**) The schematic summaries our findings such that Sca1+ cells are the most primative giving rise to CXCL12 producing stromal cells, CD146+ cells and CD166+ cells. CD146+ and CD166+ cells are osteo-progenitors. It is possible that CD146+ is an intermidiary cell fate between Sca1+ cells and CD166+ cells.

We used a *KITL* shRNA to knock down the expression of *KITL* in the Sca1^+^ cells. Using the renal sub-capsule assay, we combined fetal skeletal progenitors and Sca1^+^/shKITL cells (Fig. 7a), we found that the Sca1^+^/shKITL cells still grew in the BM area of graft (Fig. 7b). However, knockdown of *KITL* resulted in fewer LSK cells (Fig. 7c) and reduced marrow cellularity (Fig. 7d) in the graft. We conducted qRT-PCR prior to transplantation and droplet digital PCR (ddPCR) post transplantation to demonstrate that the *KITL* expression was decreased in the *KITL* shRNA treated cells versus scramble control (Supplementary Fig 8a,b). BM reconstitution assays showed that lethally irradiated mice that received marrow cells from the *KITL* knockdown graft did not survive, strongly suggesting there were few or no functional HSCs in the marrow of the graft (Fig. 7e). These results suggest that Sca1^+^ cells are an important source of KITL in the niche needed to maintain and or recruit HSCs.

## Discussion

In this study, we identified a mesenchymal stromal progenitor hierarchy with a primitive mesenchymal stromal progenitor that can give rise to two distinct osteo-progenitors in the adult BM haematopoietic niche. Their identities as niche cells were supported by four independent approaches—*in vitro* cultures, *in vivo* transplantation, *in vivo* ablation and quantitative single-cell gene expression analyses. Our data indicated that Sca1^+^ cells represent common mesenchymal stromal progenitors that can give rise to osteogenic CD146^+^ and CD166^+^ progenitors and CXCL12-producing stromal cells in the marrow (Fig. 7f). Although the lineage relationship between the CD146^+^ cells and CD166^+^ progenitors has not been determined, our gene expression analysis (Fig. 5) suggests that CD146^+^ cells represent a more primitive progenitor than CD166^+^ cells, possibly acting as an intermediary progenitor.

Our finding that Sca1^+^ and CD146^+^ both localized to perivascular region but have different differentiation potentials *in vivo* is especially important. It directly supports the notion that not all perivascular stromal cells are identical. Although several perivascular stromal cells have been implicated in HSC maintenance, the lineage relationship among them remains unclear. Leptin receptor is expressed in all three of our mesenchymal progenitors (Supplementary Fig. 6d,e). In contrast, Nestin expression varies among our progenitors. Because of low Nestin expression (Fig. 5a) and detection of very few Sca1 and nestin co-expressing cells in Nestin-GFP mouse BM (Supplementary Fig. 6a,b), we believe that our Sca1^+^ progenitors do not overlap with nestin^+^ MSCs. All of our mesenchymal progenitors produced CXCL12 (Fig. 5a), a defining characteristic of CAR cells. However, none of the progenitors fits the surface marker definition of CAR cells (CD44^+^CD51^+^CD105^+^CD140a^+^). Using an ectopic bone-forming assay and direct intravenous transplantation model, we demonstrated that Sca1^+^ progenitors developed into cells that phenotypically resembled CAR cells and produced CXCL12 (Fig. 2f and 4d and Supplementary Fig. 5a,b), suggesting that CAR cells can be derived from the Sca1^+^ progenitors. More analyses are needed to define the developmental pathway between Sca1^+^ progenitors and CAR cells in adult mice. The periosteum on the outer portion of the bone also expresses Sca1. We isolated the periosteum by scrapping and performed a CFU assay, they failed to produce any colonies and thus are unlikely to have a significant contribution to our population analysis (Supplementary Fig. 2h).

In contrast to fetal CD105^+^Thy1.1^−^ and postnatal progenitors that have the ability to initiate niche formation^7^,^25^, we found that none of the adult mesenchymal progenitors are capable of niche formation by themselves under comparable conditions, suggesting their ability is limited compared with fetal and postnatal progenitors which can initiate niche formation under minimal conditions. The niche-initiating ability may be suppressed in adult progenitors under homoeostasis conditions to reduce the chances of heterotopic ossification. Production of BMP2, an osteo-inductive cytokine that is involved in heterotopic bone formation, is lower in adult Sca1^+^ progenitors compared with fetal osteochondral progenitors (Fig. 5a). However the adult progenitors can participate in generation of niche cells in existing microenvironments and may contribute to the maintenance of the niche. Because CD146^+^ and CD166^+^ progenitors form bone after transplantation (Fig. 2b,d), they may be involved in both routine osteocyte turnover and bone regeneration when trauma or injury occurs. Under normal conditions, Sca1^+^ progenitors may contribute to the stromal component in the niche, mainly through differentiation into CXCL12-producing stromal cells (Fig. 2f and 3d and Supplementary Fig. 5a). Although Sca1^+^ progenitors did not form osteocytes immediately after transplantation (Fig. 2b), they eventually contributed to bone formation (Fig. 3f). This process may be accelerated in the event of bone fracture or injury to compensate for the loss of bone progenitors and to guarantee osteo-lineage turnover. Sca1 knock-out mice have phenotypically normal bones into adulthood. However, over time they exhibit dramatically decreased bone mass that results in brittle bones with increased susceptibility to fractures^26^, suggesting that Sca1^+^ progenitors might play a role in bone remodelling.

The HSC niche is far more complex than first reports suggest or than the *Drosophila* models indicate. The simple model of a niche where a single stromal cell type guides a single type of stem cell in the *Drosophila* germ line does not fully explain the formation of the mammalian BM niche. The heterogeneity within HSCs and haematopoietic progenitor populations suggests the possible existence of ‘micro-niches' within the BM microenvironment. Our detailed analysis of the mesenchymal stromal progenitor hierarchy provides a platform for better understanding the unique properties of each stromal subtype. Although all three mesenchymal progenitors could support multi-lineage hematopoiesis *in vitro*, their abilities to support myelopoiesis varied (Fig. 6b). LT-HSCs co-cultured with CD166^+^ cells produced fewer GM colonies (Supplementary Fig. 7a) and lower myeloid output after transplantation (Fig. 6b), indicating CD166^+^ cells may either preferentially support lymphopoiesis or suppress myelopoiesis. A similar bias was evident in the experiment done by the Suda group using LSK cells co-cultured with CD166^+^Sca1^−^ cells^27^. Recent publications from the Morrison and Link groups suggest the existence of a perivascular niche for HSCs and an endosteal niche for lymphoid progenitors^28^,^29^. It is interesting to note that Sca1^+^/CD146^+^ progenitors localized to the perivascular regions (Fig. 1d,e), whereas CD166^+^ cells mainly localized to the endosteal surface (Supplementary Fig. 1g). Moreover, canonical Wnt signalling was reported to inhibit lymphopoiesis and non-canonical Wnt signalling facilitated lymphoid commitment^30^. DKK1, a well-known canonical Wnt signalling inhibitor, is highly expressed in CD166^+^ cells (Fig. 5e). These data indicate potential mechanisms for how different stromal subsets may influence HSC fate decisions. However, more systematic and mechanistic studies are needed to define how different niche components integrate their activity to provide physiological support for different HSCs and haematopoietic progenitors.

By knocking down KITL expression in transplanted Sca1^+^ progenitors, we directly demonstrated that Sca1^+^ progenitors and their progeny are not merely structural components, but they actively provide functional support to HSCs in the niches (Fig. 7). The impaired ability of KITL knockdown Sca1^+^ cells to recruit or support hematopoiesis was reflected in the lower LSK and total cell number harvested from the ectopic niche (Fig. 7c,d). Our data indicate that perivascular Sca1^+^ progenitors are an important source of the KITL, consistent with results from the Morrison group^23^. However our results do not exclude the possibility that KITL also plays a role in maintenance and or differentiation of the mesenchymal stromal cell population. Mechanistic studies into the specific role of KITL in the Sca1+ stromal cell population are of future interest and would enhance the understanding of the HSC niche. Collectively, our results provide convincing evidence that perivascular Sca1^+^ cells function as a common progenitor of niche components in the murine adult BM.

## Methods

### Mice

C57BL/Ka (CD45.2), C57BL/Ka-CD45.1, C57BL/Ka-Thy1.1-CD45.1, C57BL/Ka- Thy1.1-CD45.1-/Tg(CAG-GFP) and B6-Rag-2^−/−^γc^−/−^ (CD45.2) mice were maintained by the Animal Resource Center of City of Hope under SPF conditions. Timed embryos from C57BL/Ka-Thy1.1-CD45.1 mice were used in the fetal skeletal progenitors/adult mesenchymal progenitor co-transplantation studies. B6-Rag-2^−/−^γc^−/−^ mice that were 8–12 weeks old were used as recipients for fetal bone transplantation. C57BL/Ka or C57BL/Ka-CD45.1 mice were used as recipients in HSC and stromal cell transplantation assays. Flk1-GFP, Osx1-Cre:GFP, C57BL/6-tdTomato, UBC-CreERT2 and iDTR mice were obtained from Jackson Labs. The Col2.3-GFP mice were a kind gift of Dr Ravi Bhatia. The nestin-GFP mice were kindly provided by Dr Qiang Lu^31^. The CD166-GFP mice^32^ were a generous gift from Dr Ya-Huei Kuo. Mouse care and experimental procedures were performed in accordance with federal guidelines and protocols approved by the Institutional Animal Care and Use Committee at City of Hope.

### Isolation of adult mesenchymal progenitors

Tibias and femurs were excised from eight to 12-week-old mice. After cleaning the muscle and connective tissue, the bones were crushed gently with a mortar and pestle in PBS buffer. The crushed mass was washed with PBS to remove all of the BM cells. The remaining bone fragments were incubated at 37 °C with collagenase I (3 mg ml^−1^; Sigma) and gently agitated for 60 min. The digested bones were then filtered through a 40 μm strainer (BD Bioscience), pelleted at 200*g* at 4 °C, and resuspended in PBS. The marrow cells were collected and red blood cells were depleted with ACK lysis buffer. The cells were blocked with an anti-CD16/32 antibody and stained with labelled monoclonal antibodies against CD45 (1:100 BioLegend 103128), CD31 (1:100 BioLegend 102418), Ter119 (1:500 BioLegend 116210), Sca1 (1:20 BioLegend 108133), CD146 (1:25 BioLegend 134705) and CD166 (1:25 R&D Systems FAB1172P) or antibodies against CD45, CD31, Ter119, CD51(1:100 BioLegend 104105), CD105 (1:100 BioLegend 120410) and Thy1.1 (1:100 BioLegend 140306). The stained cells were then sorted using a FACSAria III sorter (BD Bioscience). The purity of sorted cells was confirmed by flow cytometry to be greater than 99%. All antibodies were purchased from Biolegend or eBioscience unless otherwise noted.

### Isolation of LT-HSCs

Haematopoietic stem and progenitor cells were enriched from RBC-lysed BM cells using the c-Kit microbeads kit (Miltenyi Biotec) following the manufacture's instructions. The enriched haematopoietic stem and progenitor cells were then blocked with anti-CD16/32 antibody, stained with labelled antibodies against c-Kit (1:100 BioLegend 105826), Sca1 (1:100BioLegend 108114), CD150 (1:100 BioLegend 115912), CD48 (1:100 BioLegend 103414), CD135 (1:50 BioLegend 135306) and lineage (CD3 1:100 , CD4 1:200, CD8 1:100, CD11b 1:200, B2201:200, Gr-1 1:200, Ter119 1:100 [all Biolegend]), then sorted using a FACSAria III. The LT-HSCs were defined using the following cell-surface markers: lineage-Sca1^+^c-Kit^+^CD150^+^CD48^−^CD135^−^.

### Stromal cell culture and CFU-F assay

All stromal cells were cultured in MEM-alpha medium (Gibco) containing 20% fetal bovine serum (FBS; Atlanta Biologicals) at 37 °C with 5% CO_2_. For single-cell CFU-F assays, single stromal cells were sorted using a FACSAria III into 96-well flat bottom plates containing MEM-alpha medium. The colonies were counted after 2 weeks using an inverted microscope (Leica).

### LT-HSC and stromal cell *in vitro* co-culture assay

Bone-disassociated stromal cells (5,000) from CD45.2 mice and CD45.1^+^ LT-HSCs (1,000) were cultured in IMDM medium (Gibco) containing 10% FBS, 1 ng ml^−1^ KITL (Peprotech) and 1 ng ml^−1^ TPO (Peprotech) for 2 days. Harvested CD45.1^+^ HSCs were either sorted individually into 96-well U bottom plates for the single-cell CFU assay^33^ or transplanted into lethally irradiated (1,100 rads) CD45.2 recipient mice (250 cells/mouse initial seeding HSC number) together with 200,000 CD45.2^+^ helper marrow cells. For the CFU assay the single cells were cultured in IMDM medium (Gibco) containing 10% FBS, 10ng ml^−1^ of the following cytokines (Peprotech) IL-1a, IL-3, IL-5, IL7, IL-9, IL-10, IL-11, GM-CSF, TPO, EPO, SCF and Flt3, colonies were counted 7 days after seeding. For the transplantation assay. For the transplantation assay, peripheral blood was drawn at the indicated time points and the contribution of donor-derived cells was analysed monthly by flow cytometry.

### Transplantation of adult mesenchymal progenitors

The kidney capsule transplantation procedure was performed as follows: 5,000 sorted adult stromal cells alone or with 30,000 unsorted fetal bone cells were mixed with 5 μl of matrigel and injected underneath the renal capsule of 8–12-week-old B6-Rag-2^−/−^γc^−/−^ mice. The grafts were harvested 1 month later and imaged using a Leica M205 FA stereomicroscope. Alternatively, 5,000 sorted adult stromal cells in 100 μl PBS were injected intravenously into irradiated 8–12-week-old mice (600 rad). Long bones of recipient mice were harvested 1 month after transplantation.

### Tissue section and immunofluorescence staining

Freshly dissected long bones and kidney grafts were fixed in 4% paraformaldehyde for 30 min at 4 °C then decalcified in 10% EDTA at 4 °C for 5–7 days. The bones were then embedded in 10% gelatin in PBS, frozen on dry ice and stored at −80 °C. Bones were sectioned using the CryoJane taping system (Instrumedics). Sections were blocked with 1% BSA, then probed with primary antibody at 4 °C overnight; purified rat-anti-Sca1 (1:50 BioLegend 108102), purified rat-anti-CD146 (1:50, BioLegend 134702), CD166-Fitc (1:25 R&D Systems FAB1172F), CD45-Alexa647 (1:50 BioLegend 103124) and/or rabbit-anti-laminin (1:200 Sigma-Aldrich L9393-.2ML). After washing with PBS, the sections were probed with Alexa dye-conjugated antibodies, Invitrogen goat anti-rat-555 or anti-rat-488 and anti-rabbit-555 (Alexa antibodies used at 1:100), then washed and stained with Dapi and mounted in ProLong Gold anti-fade mounting media. Sections were analysed using an Olympus IBX81 microscope. Slides that passed this initial inspection were scanned using a Zeiss LSM 700 confocal microscope or iCys Research imaging cytometer (iCys, CompuCyte). Zen 2012 and Amira 3D Projection and Analysis software were used to create the maximum intensity projections and three-dimensional surface renderings of confocal *z*-stacks, respectively.

### Single-cell multi-gene quantitative real-time PCR

We double sorted individual stromal cells into 10 μl SuperScript III One-Step RT-PCR System with Platinum *Taq* DNA polymerase (Invitrogen) pre-amplification mixture made with the TaqMAN Gene expression assays in Supplementary Table 1, then reverse transcribed and amplified under the following parameters 15 min at 50 °C, (2 min 95 °C, 4 min 60 °C) for 22 cycles, 15 min at 4 °C. The resulting amplified cDNAs were diluted 1:4 and analysed by Q-PCR using the Fluidigm Biomark Dynamic Array following the manufacturer's instructions. The data were processed using the Biomark system software suite and analysed/normalized using the Fluidigm SINGuLAR Toolset 3.0. Outliers were determined using the built-in outlier identification function within the SINGuLAR Toolset and principal component analysis. The TaqMan Gene Expression Assays (Applied Biosystems) assays used are listed in Supplementary Table 1.

### Quantitative real-time PCR

RNA was extracted from sorted cells using an RNeasy RNA isolation kit (Qiagen) then reverse transcribed into cDNA with the Sensiscript RT kit (Qiagen). The cDNA was amplified for specific targets using TaqMan in a ViiA 7 Real-Time PCR System (Life Technologies). The thermal cycling parameters were 40 cycles of 95 °C for 15 s and 60 °C for 1 min. Data were analysed with the 7500 Fast System SDS software. The TaqMan assays used are listed in Supplementary Table 1.

### Droplet Digital PCR (ddPCR)

Sorted GFP+/− cells from kidney grafts were collected for RNA isolation and cDNA synthesis, as above. An amount of 1 ul cDNA was mixed with 2 × concentrated ddPCR Supermix for Probes (Cat. 186-3026, BioRad) and Taqman probe for KITL and GAPDH (Applied Biosystems) according to manufacturer's protocol. Droplets were generated with 20 μl PCR reaction mix and 70 μl oil in a DG8 cartridge using QX 200 droplet generator (Bio-Rad). The resulting droplets were then transferred into a PCR plate. After PCR amplification, the plate was read using a QX200 droplet reader to provide absolute quantification of the target genes. QuantaSoft software (Bio-Rad) was used to analyse the positive counts per sample. The counts of target genes (copies/ul reaction) for each sample were normalized to internal GAPDH control.

### shRNA transduction

KITL-specific shRNA knockdown constructs^7^ using pll3.7 shRNA cloning vector and active lentiviral stocks were generated as previously described^34^, using the VSV.G and CMVDR8.74 packaging plasmids along with jetPRIME transfection reagent (manufactures protocol). The sorted, adult bone-disassociated Sca1^+^ cells were resuspended in MEM-alpha medium with 15% fetal calf serum (FCS) and transduced with shRNA lentiviral vectors carrying the KITL-specific shRNA or a scrambled control. Forty-eight hours after transduction, 1,000 GFP^+^ cells were sorted and transplanted under the kidney capsule with 20,000 fetal skeletal progenitors in 5 μl matrigel. The grafts were harvested 1 month later for analysis.

### Statistical Analysis

All data are presented as mean±s.d. and two group comparisons were performed using a two-tailed unpaired Student's *t*-test. Analysis of variance analysis was conducted on the single-cell qRT-PCR gene expression data, while only 18 cells are shown on the heatmap they are representative of several repeats that amounted to 72 individual cells per group and were used for the one-way analysis of variance analysis. Survival was measured using Kaplan–Meier analysis. A value of *P*≤0.05 was considered statistically significant for all tests. Statistical test were not used to determine the sample size. No randomization was used to allocate animals to particular groups; age and sex-matched recipients were used for transplantation experiments. The investigators were not blinded to experimental groups during analysis. Statistical analyses were performed using GraphPad Prism software v.6.

### Data Availability

The authors declare that all data supporting the findings of this study are available within the article and its Supplementary Information files or from the corresponding author upon reasonable request.

## Additional information

**How to cite this article**: Hu, X. *et al*. Identification of a common mesenchymal stromal progenitor for the adult haematopoietic niche. *Nat. Commun.* **7**, 13095 doi: 10.1038/ncomms13095 (2016).

## Supplementary Material

Supplementary Information Supplementary Figures 1-8, Supplementary Table 1.
