# Supplementary material for: Identification of a common mesenchymal stromal progenitor for the adult haematopoietic niche
Source: Nat Commun. 2016 Oct 10;7:13095. doi: 10.1038/ncomms13095 (PMC5062560; doi:10.1038/ncomms13095)
Supplement: Supplementary Information — Supplementary Figures 1-8, Supplementary Table 1. [file ncomms13095-s1.pdf]

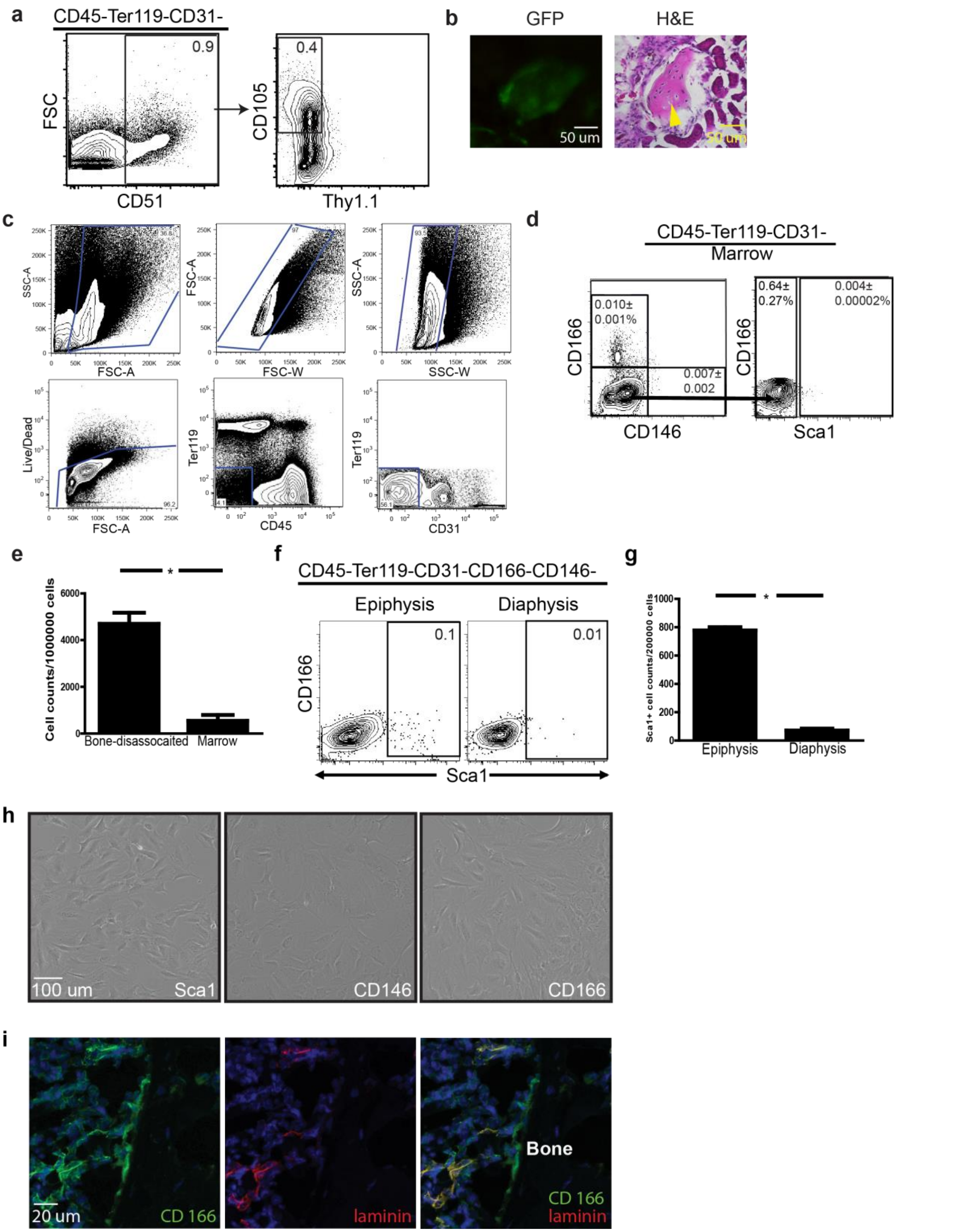

Supplementary Figure 1. Adult mesenchymal progenitors can not initiate niche formation and most Sca1⁺ cells localize to the epiphysis. (a) Representative FACS profiles of stromal

cells ($CD45^-Ter119^-CD31^-$). They were separated based on the same markers that identified the fetal osteochondroprogenitors: CD51, CD105 and Thy1.1. The number shown in the gate is the percentage of total live cells. (b) GFP-labeled $CD105^+Thy1.1^-$ adult progenitors one month after subrenal capsule transplant (left). A representative cross-section of the graft site was stained with H&E (right; arrowhead points to bone). (c) Representative gating used to isolate the Ter119-CD45-CD31- stromal cells (d) Representative FACS profiles of stromal cells ($CD45^-Ter119^-CD31^-$) from the marrow compartment (e) $Sca1^+$ cell distributions within bone-disassociated and marrow fractions (*:$P<0.05$, n=6, student's t-test). (f) Representative FACS analysis of $Sca1^+$ frequency in stromal cells from the diaphysis and epiphysis that were pre-gated for $CD45^-Ter119^-CD31^-CD166^-CD146^-$. (g) $Sca1^+$ cell counts from the diaphysis and epiphysis (*:$P\leq0.05$, n=6, student's t-test). (h) Bright-field image of cultured mesenchymal progenitors. (i) Confocal image of cells stained with $CD166^+$ and laminin antibodies.

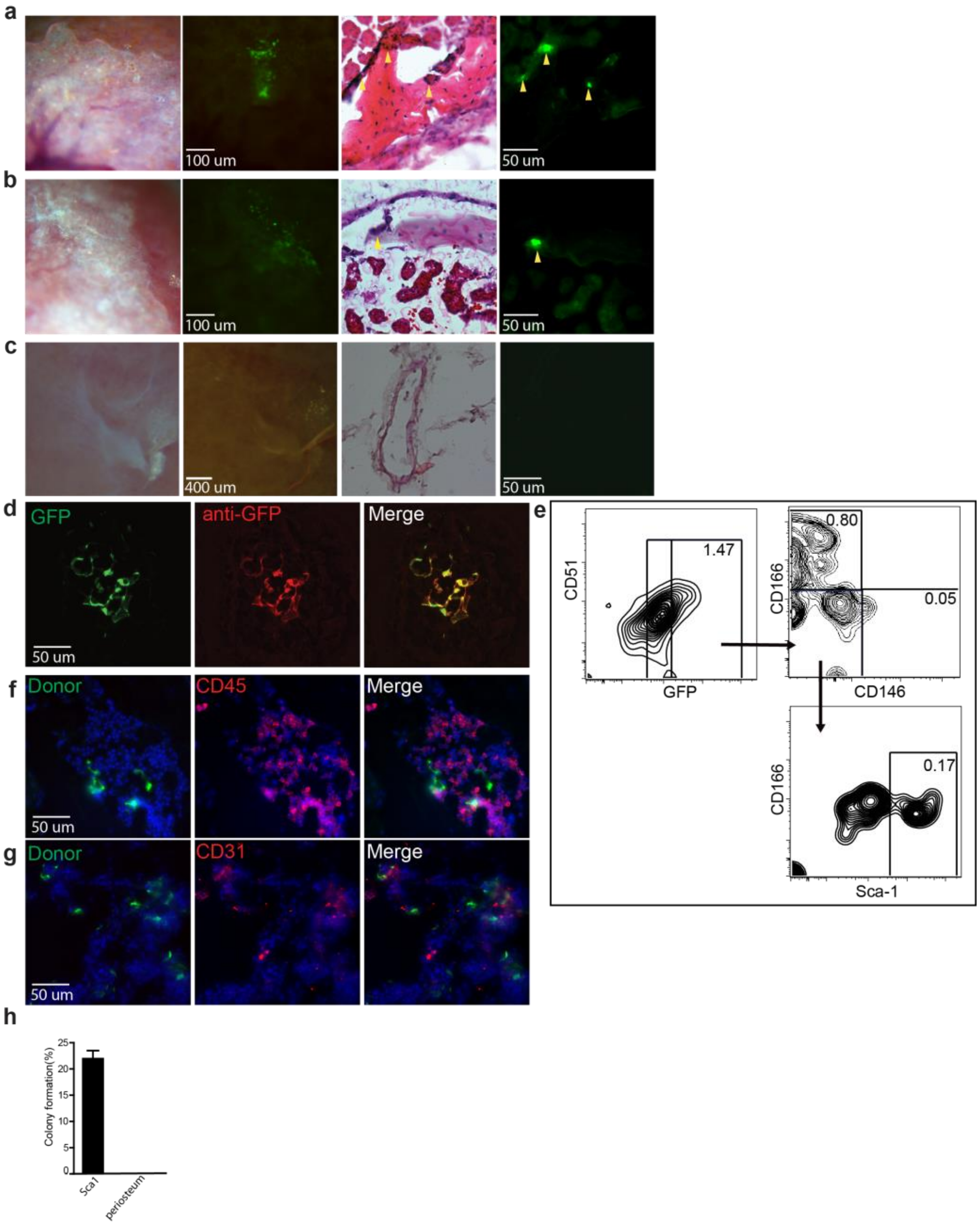

Supplementary Figure 2. Sca1^{+} progenitors did not generate progeny with hematopoeitic or endothelial fate (a-c) Bright field and GFP images of GFP-labeled adult progenitors (a) CD146^{+} (b) CD166^{+} (c) Sca1^{-} mixed with fetal skeletal progenitors one month after transplant. Donor-derived GFP^{+} cells can be clearly identified (far left and left). Representative cross sections of the graft site stained with H&E (right) or GFP (far right) to identify donor origin. (d) Cross section of a representative graft derived from GFP labeled Sca1^{+} progenitors co-transplanted with fetal skeletal progenitors. GFP is shown in green and anti-GFP antibody staining is shown in red. (e) Representative gating for the GFP cells with the highest intensisity of GFP. The percent of live cells is displayed. (f and g) GFP labeled Sca1^{+} progenitors were co-transplanted with unmarked fetal skeletal progenitors under the kidney capsule. Engrafted bones were harvested one month later and sections were stained with an antibody to (f) CD45 or (g) CD31. (h) Cell colony forming efficiency of Sca1+ bone-disassociated sorted cells and unsorted periosteum from the outer bone.

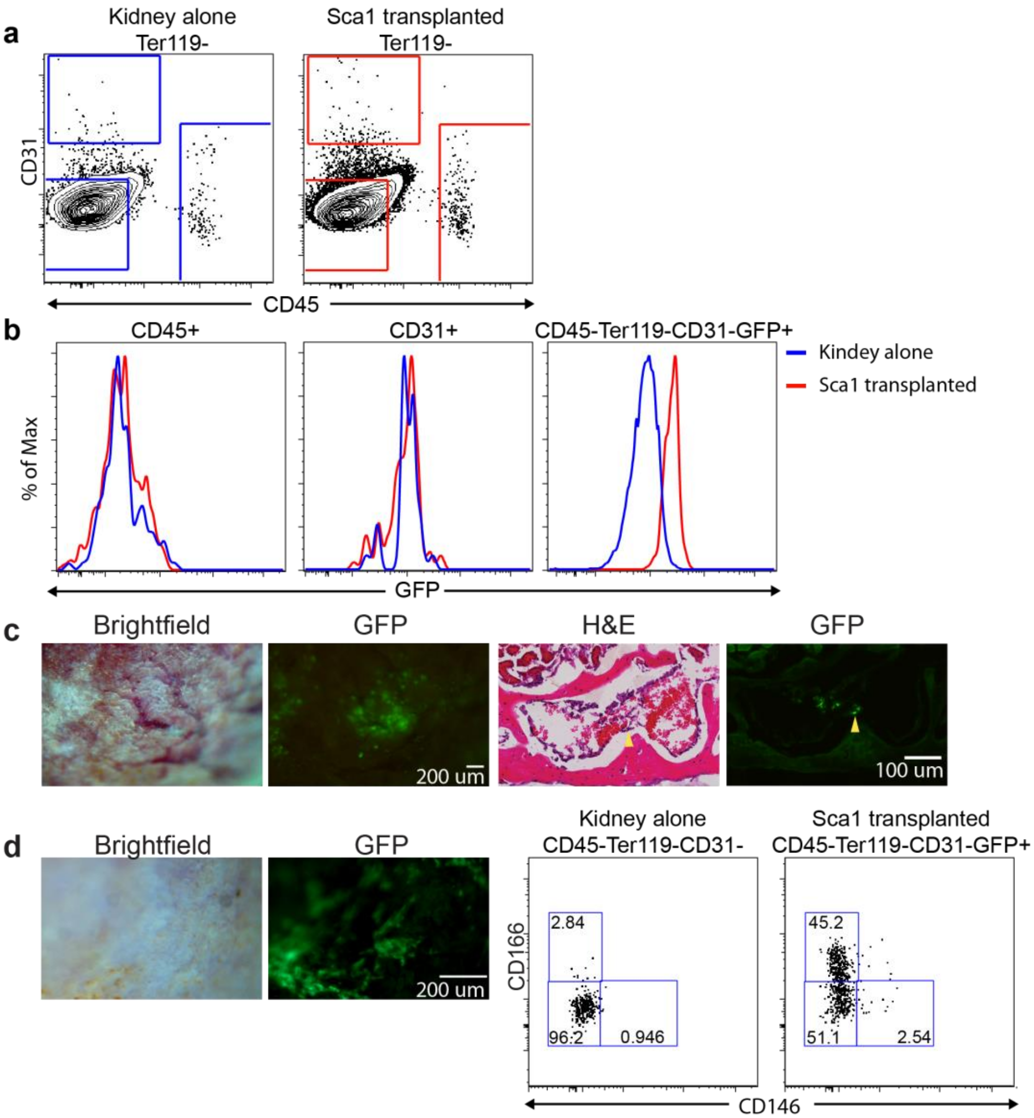

Supplementary Figure 3. GFP is restricted to Sca1$^+$ transplanted cells and its progeny. A single Sca1$^+$GFP$^+$ clone can give rise to multiple cell types. (a) Representative FACS plots of a untransplanted kidney and a kidney transplanted with Sca1$^+$GFP$^+$ cells, showing gating for stromal cells (CD45$^-$Ter119$^-$CD31$^-$), hematopietic cells (CD45$^+$) and endothelial cells (CD31$^+$). (b) Representative histogram of GFP expresssion in hematopoietic (CD45$^+$), endothelial cells (CD31$^+$) and stromal cells (CD45$^-$Ter119$^-$CD31$^-$GFP$^+$) from an untransplanted kidney (blue) and a kidney transplanted with Sca1$^+$GFP$^+$ cells. (c) A single sorted Sca1$^+$ cell was expanded *in vitro* for two days then transplanted with unmarked fetal skeletal progenitors under the kidney capsule. Bright field and GFP images of GFP-labeled progenitors one month after transplant (far left and left). A representative cross section of the graft site was stained with H&E (right) or GFP to identify the donor origin (far right). Yellow arrows show donor-derived cells. (d) A single sorted Sca1$^+$ cell was expaned *in vitro* for 10 days then transplanted alone under the kidney capsule. Bright field and GFP images are shown (far left and left). Representative FACS analysis of the kidney from an untransplanted mouse and a kidney trasplanted with the Sca1$^+$ clone (right and far right). Prominent CD166 and CD146 populations can be found in the Sca1$^+$ clone tranplanted kidney (far right).

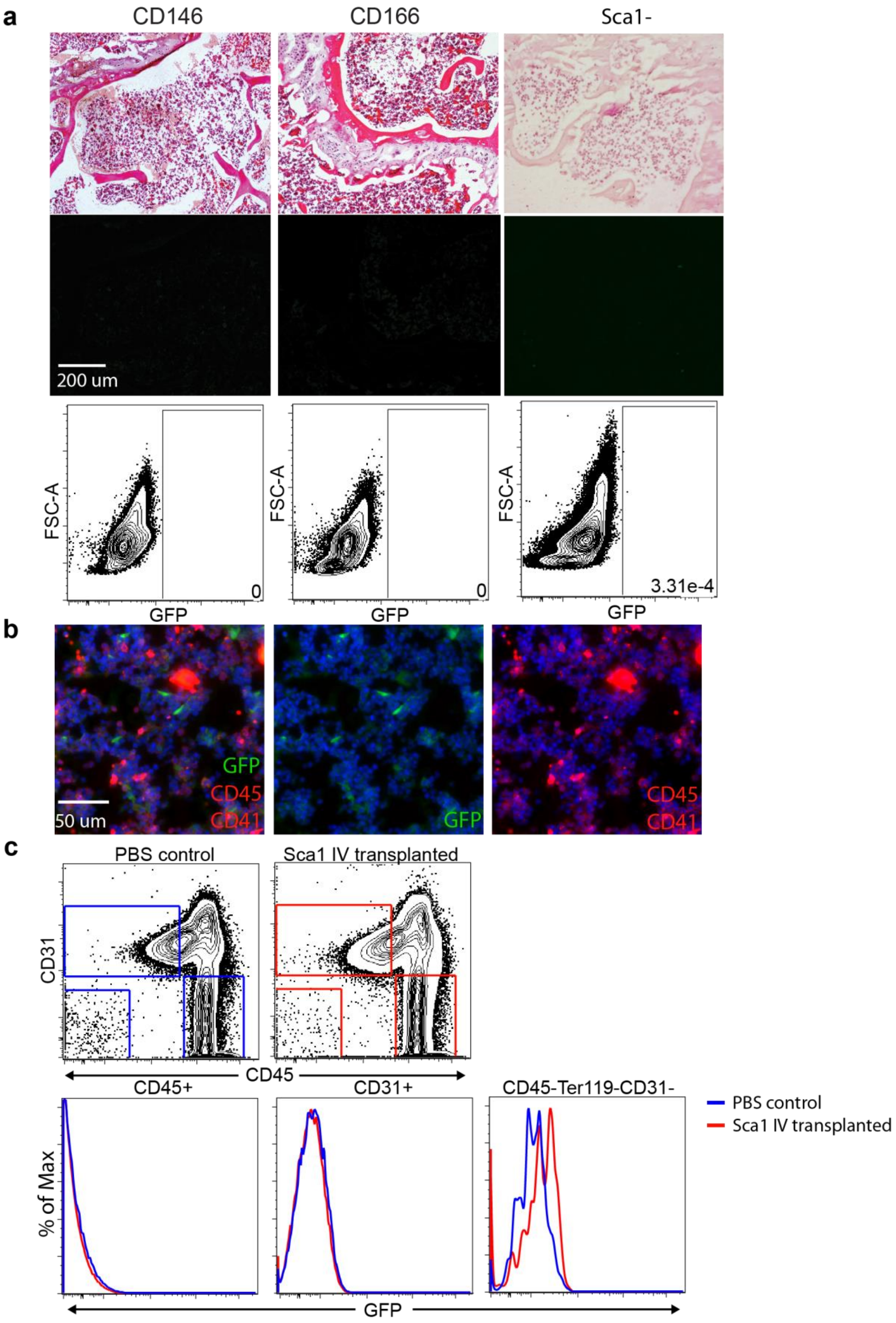

a
CD146
CD166
Sca1-
200 um
FSC-A
GFP
0
0
3.31e-4
b
GFP
CD45
CD41
50 um
c
PBS control
Sca1 IV transplanted
CD31
CD45
CD45+
CD31+
CD45-Ter119-CD31-
% of Max
GFP

Supplementary Figure 4. Sca1+ progenitors retain GFP expression and do not give rise to hematopoietic or endothelial cells. (a) Representative cross sections of the tibia stained with H&E (upper) or GFP (middle). Tibias were harvested one month after transplantation. Representavie FACS analysis of GFP expression in femurs harvested one month after transplantation (lower). (b) Representative crossection from mouse one month after IV transplantation of Sca1$^+$ cells stained with CD45 and CD41 antibodies (c) Representative FACS plots of a PBS control and mouse transplanted with Sca1$^+$GFP$^+$ cells, showing gating for stromal cells (CD45$^-$Ter119$^-$CD31$^-$), hematopietic cells (CD45$^+$) and endothelial cells (CD31$^+$) (upper). Representative histogram of GFP expresssion in hematopoietic (CD45$^+$), endothelial cells (CD31$^+$) and stromal cells (CD45$^-$Ter119$^-$CD31$^-$) from an PBS cotnrol (blue) and Sca1$^+$GFP$^+$ IV transplanted mouse. Femurs were harvested one month after IV transplantation (lower).

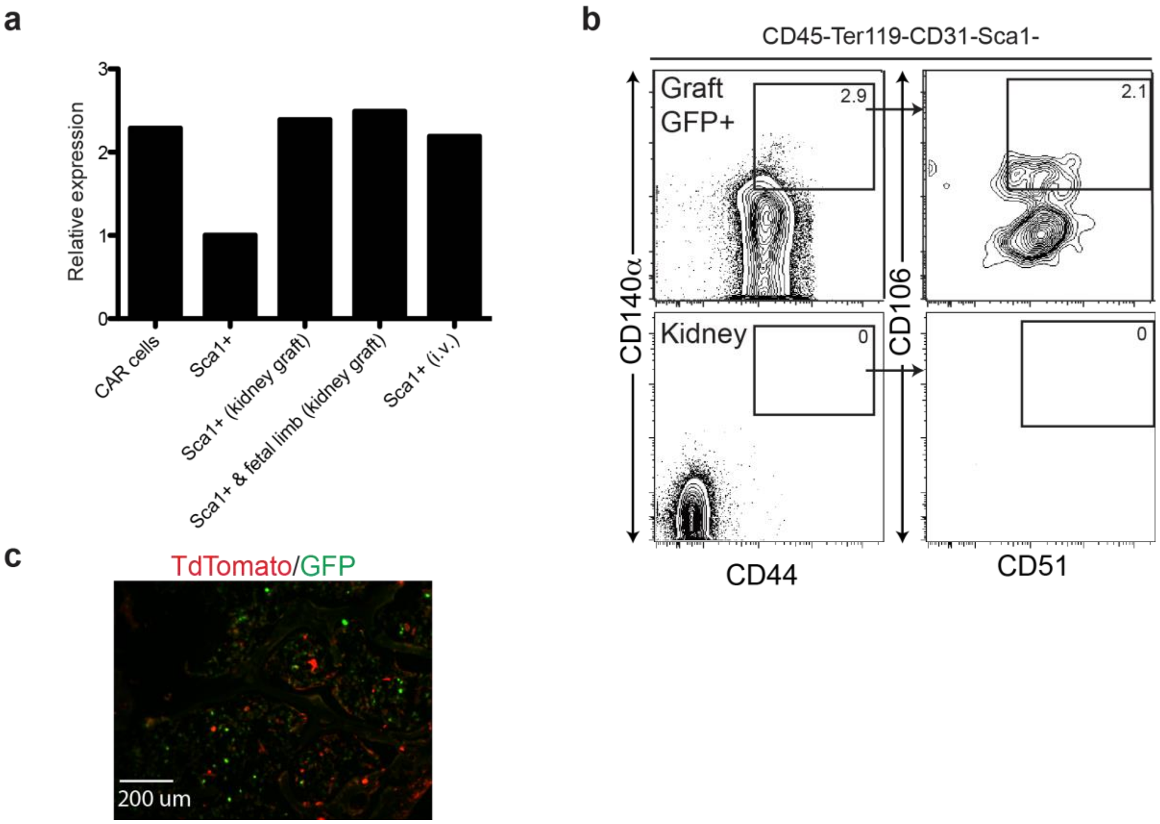

Supplemntary Figure 5. Sca1$^+$ progenitors and their progeny produced CXC12 and do not fuse with host cells. (a) Relative CXCL12 expression levels in sorted marrow CAR cells, Sca1$^+$ progenitors and GFP$^+$ cells that were derived from Sca1$^+$ progenitors after transplant and analysed by qRT-PCR normalized to GAPDH. (b) FACS analysis of phenotypically defined CAR cells in the kidney and in the graft arising from the direct transplant of GFP-labeled Sca1$^+$ progenitors under the kidney capsule one month after transplant. (c) GFP$^+$ Sca1$^+$ progenitors were intravenously injected into irradiated B6-TdTomato mice (600 rads). No donor-host fusions were deteced one month later.

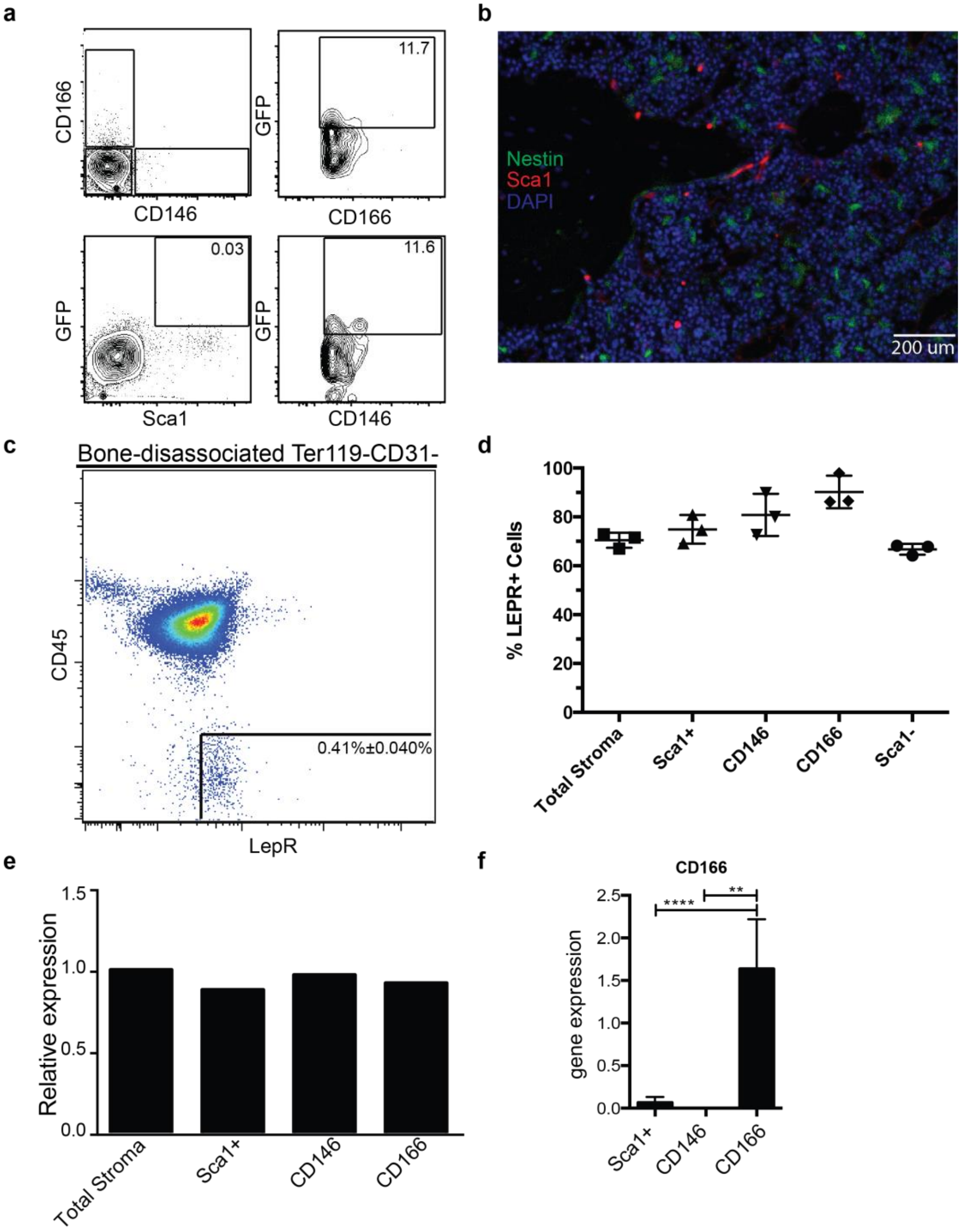

Supplementary Figure 6. Sca1$^+$ progenitors expressed leptin receptor but little or no nestin. (a) FACS analysis of bone-disassociated stromal cells (CD45$^-$TER119$^-$CD31$^-$) from nestin-GFP mice. (b) Sections of femurs from nestin-GFP mice stained with an antibody to Sca1. (c) Representative FACs analysis of LepR$^+$ cells showing frequency in bone-disassociated live cells, n=3. (d) Frequency of LepR+ cells in bone-disassociated total stroma, Sca1$^+$, CD146$^+$, CD166$^+$ and Sca1$^-$, n=3. (e) Relative *LepR* receptor expression levels in sorted total bone-disassociated stroma, Sca1$^+$, CD146$^+$ and CD166$^+$ progenitors analysed by qRT-PCR normalized to GAPDH. (f) Gene expression of CD166 in the sorted stromal cells based on single-cell Q-PCR (**:P<0.005, ***P<0.0005), n=72, student's t-test).

.

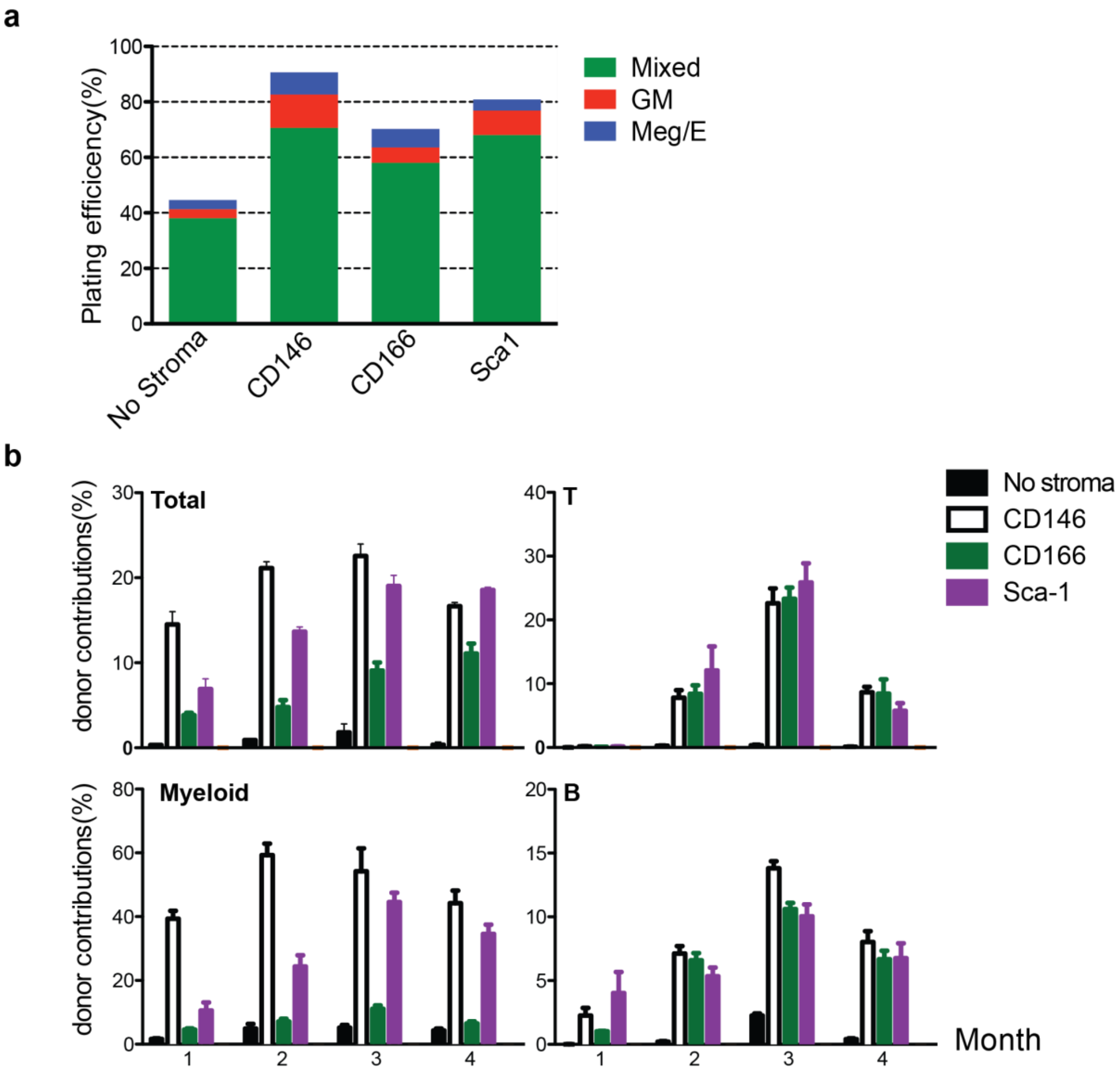

Supplementary Figure 7. *In vitro* expanded bone-disassociated mesenchymal progenitors retain the ability to support hematopoesis. (a) Single cell colony forming effciency of LT-HSCs co-cultured with different mesenchymal progenitors (n=180). (b) LT-HSCs were cultured with *in vitro* expanded mesenchymal progenitors for two days then tested in a competitive repopulation assay. The contribution of donor HSCs in the blood assayed one to four months after transplant (n=8).

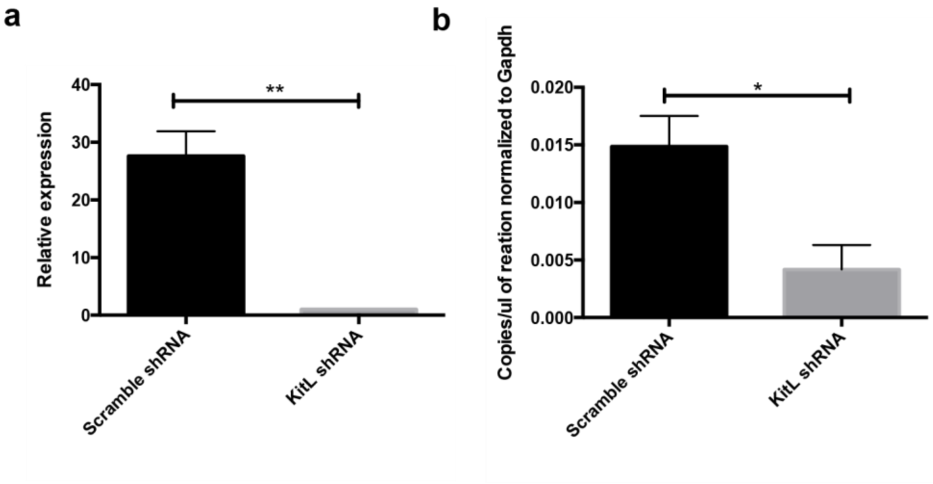

Supplemenatry Figure 8. KitL shRNA effectively supressed KitL expression in Sca1+ cells pre- and post transplantation. (a) Relative expression of *KITL* in Sca1+ cells transduced with scramble or *KITL* shRNA, measured by standard Taqman assay qRT-PCR, prior to transplantation into the kidney capsule. (b) Copy number of *KITL* per ul of ddPCR reaction of Scramble or *KITL* shRNA transduced Sca1 cells 1 month after kidney capsule transplantation, (*:P<0.05, **P<0.005, technical triplicates, student's t-test)

Supplementary Table 1. TaqMan probes used in current study.

Gene	TaqMan Assay ID
ALP	Mm00475834_m1
Angpt1	Mm00456503_m1
Angpt2	Mm00545822_m1
BMP2	Mm01340178_m1
BMP4	Mm00432087_m1
BMP6	Mm01332882_m1
BMPR1α	Mm00477650_m1
CaSR	Mm00443375_m1
Cathepsin K	Mm00484039_m1
CD105	Mm00468256_m1
CD140β	Mm00435546_m1
CD166	Mm00711623_m1
CD44	Mm01277163_m1
CDH11	Mm00515466_m1
CDH2	Mm00483213_m1
CXCL12	Mm00445553_m1
M-CSF	Mm00432686_m1
GM-CSF	Mm01290062_m1
G-CSF	Mm00438334_m1
DKK1	Mm00438422_m1
FGF2	Mm00433287_m1
FGF4	Mm00438917_m1
FGF7	Mm00433291_m1
FLT-3 L	Mm00442801_m1
GAPDH	Mm99999915_g1
ICAM1	Mm00516023_m1
IGF-1	Mm00439560_m1
IGFBP2	Mm00492632_m1
IL-1	Mm01336189_m1
IL-10	Mm00439614_m1
IL-3	Mm00439631_m1
IL-6	Mm00446190_m1
IL-7	Mm01295803_m1
Leptin receptor	Mm00440181_m1
KITL	Mm00442972_m1
MMP9	Mm00442991_m1
NCAM1	Mm00580526_m1
Nestin	Mm00450205_m1
Osteocalcin	Mm03413826_mH
Osteopontin	Mm00436767_m1
Osterix	Mm00504574_m1
TGFβ1	Mm01178820_m1
TIMP3	Mm00441826_m1
TNFα	Mm00443258_m1
TPO	Mm00456355_m1
VCAM	Mm01320970_m1
VEGF	Mm01281449_m1
Wnt3a	Mm00437337_m1
Wnt5a	Mm00437347_m1
